# Mechanistic Insight into Conformational Control of Enzyme Activity by Genetically Encoded Metal‐Responsive Switches

**DOI:** 10.1002/cbic.70349

**Published:** 2026-04-24

**Authors:** Payal, Jonathan Thirman, Katherine A. Edmonds, Sandip Mishra, Nathan Blackwell, Yasmine S. Zubi, Benoît Roux, Jared C. Lewis

**Affiliations:** ^1^ Department of Chemistry Indiana University Bloomington Indiana USA; ^2^ Department of Biochemistry and Molecular Biology University of Chicago Chicago Illinois USA

**Keywords:** artificial metalloenzyme, conformational switching, prolyl oligopeptidase, unnatural amino acid

## Abstract

We previously introduced a genetically encoded, metal‐responsive system for reversible control of protein function based on metal chelation by bipyridylalanine (BpyAla) residues. The efficacy of this linking group approach was demonstrated in two structurally and functionally distinct enzymes, *Pyrococcus furiosus* prolyl oligopeptidase (*Pfu* POP) and *Photinus pyralis* luciferase (Pluc). Here, we investigate the mechanistic basis of this switching in *Pfu* POP. Fluorescence‐based metal competition assays and molecular dynamics (MD) simulations were conducted to quantify Ni(II) binding affinity and evaluate the structural response to Bpy_2_Ni(II) complex formation. ^19^F NMR spectroscopy and MD simulations further indicate that linking group‐controlled conformational changes near the catalytic triad, particularly within the loop containing H592, drive the observed activity modulation upon metal binding. These findings establish that genetically encoded metal‐binding motifs can regulate enzyme function through subtle, localized conformational changes, providing a versatile platform for engineering responsive protein systems in synthetic biology, biosensing, and programmable catalysis.

## Graphical abstract

We previously developed a genetically encoded metal‐responsive system using bipyridylalanine (BpyAla) to reversibly control protein function. Here we probe the mechanism in *Pyrococcus furiosus* prolyl oligopeptidase. Metal‐competition assays, ^19^F NMR spectroscopy, and molecular dynamics show Ni(II)‐mediated BpyAla_2_ coordination induces localized conformational changes near catalytic residue H592, modulating activity and demonstrating a general strategy for engineering metal‐regulated enzymes through subtle structural switching.

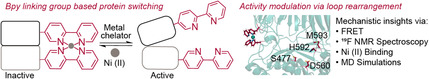

## 1 Introduction

Precise control of protein conformation is fundamental to regulating biological activity. At physiological temperatures, thermal fluctuations allow proteins to explore a range of conformational states, providing intrinsic flexibility and the potential for dynamic responsiveness [1, 2]. Many proteins have evolved stimulus‐responsive conformational switching mechanisms that regulate these motions and thus activity [3, 4]. For example, hemoglobin undergoes a well‐characterized conformational change in response to oxygen binding, which alters its affinity and enables cooperative binding of subsequent oxygen molecules [5]. Similarly, kinases adopt active or inactive conformations depending on phosphorylation states or interactions with regulatory partners, allowing precise regulation of signaling pathways [6, 7]. These conformational changes can range from subtle local rearrangements to large‐scale domain movements and are essential across diverse functional classes, from signal transduction to molecular transport [8, 9].

Inspired by such natural systems, researchers have sought to engineer synthetic protein switches that harness controlled conformational changes to modulate activity in response to specific inputs [9, 10, 11, 12, 13, 14]. These synthetic switches have found applications in biosensing [15, 16], programmable catalysis [17], targeted therapeutics [18], and biological transistors [19], enabling precise, on‐demand control of protein function. One strategy for designing protein switches involves modifying existing natural protein switches that respond to new stimuli [13]. For example, allosteric transcription factors like *E. coli* LacI have been reengineered to recognize and respond to non‐native sugar inducers beyond their natural ligands [13, 20]. Researchers have also designed chimeric systems to regulate the function of the protein of interest (POI) [21]. One such approach is optical regulation, which enables precise spatiotemporal control of POI function [14, 22]. In this approach, a natural light‐responsive domain such as the light‐oxygen‐voltage (LOV) domain of plant phototropins is fused to the POI, allowing blue light‐induced conformational changes to modulate POI function [23].

**FIGURE 1:**
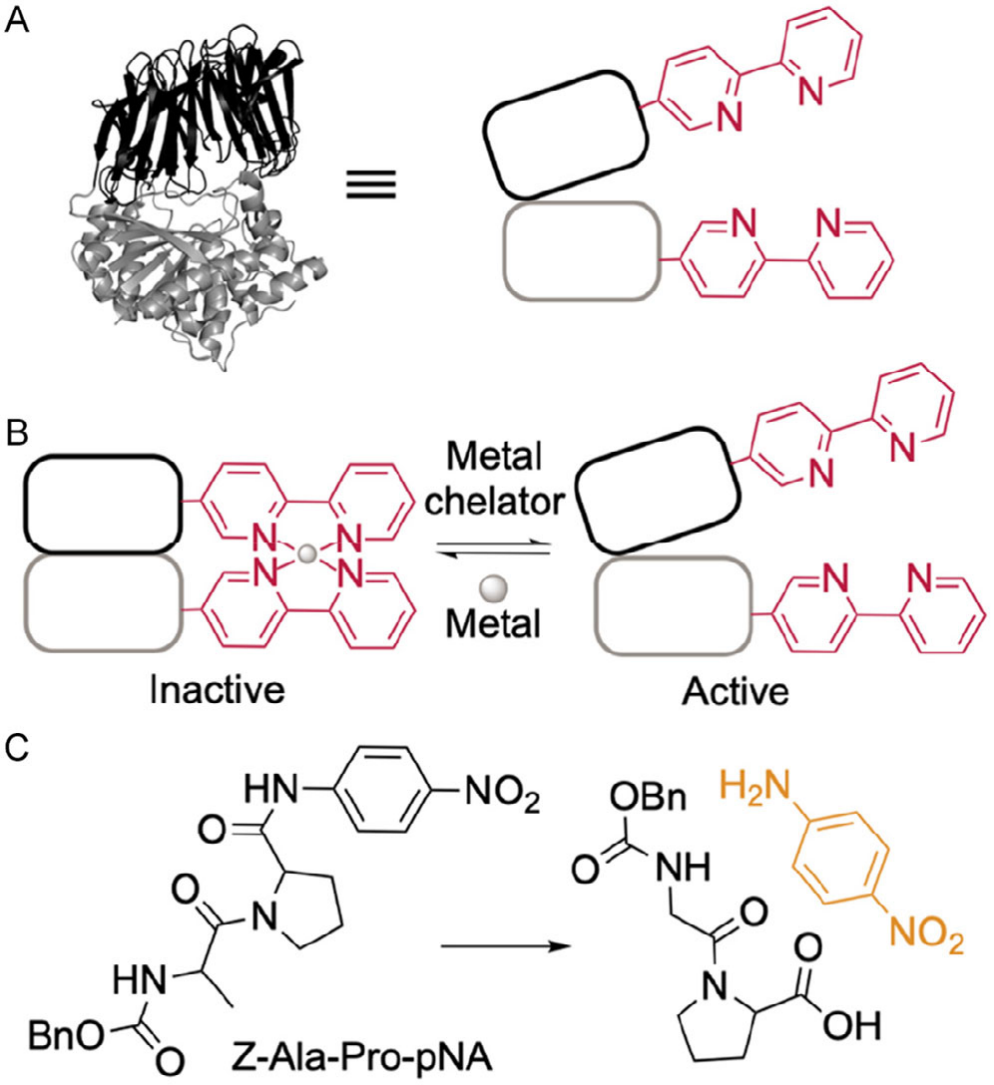
Metal‐responsive control of *Pfu* prolyl oligopeptidase (POP) activity using genetically encoded bipyridylalanine (BpyAla) linking groups [12]. (A) Structural and schematic representation of POP_Bpy2_. (B) Reversible conformational switching via M(Bpy)_2_ cross‐link formation. (C) POP activity assay involving hydrolysis of Z‐Ala‐Pro‐pNA to generate para‐nitroaniline (pNA), a chromogenic product detectable by absorbance at 410 nm.

We previously developed a strategy employing bipyridine (Bpy)‐based linking groups (LGs) that enable reversible, metal‐responsive control of protein function via formation of Bpy_2_M cross‐links between specific sites in a POI (Figure 1) [12]. These small LGs are genetically encoded, eliminating the need for extensive evolution or incorporation of additional regulatory domains and minimizing structural disruption. Molecular dynamics simulations guided selection of sites appropriate for Bpy incorporation, streamlining experimental design. Using this approach, we engineered robust, reversible switching in *Pyrococcus furiosus* prolyl oligopeptidase (*Pfu* POP) and further demonstrated Bpy LG generality by applying this strategy to a structurally and functionally unrelated enzyme, *Photinus pyralis* luciferase (Pluc).

Preliminary analysis of Bpy‐based switching in POP in the presence of Ni(II) was consistent with the formation of the desired intramolecular Bpy_2_Ni(II) crosslink. We hypothesized that this cross‐link at the interface of the two domains comprising POP could regulate its activity by eliminating domain opening and closing motions required for catalysis (Figure 1); inhibition of large‐scale motions required for Pluc catalysis could also be the source of switching in that enzyme. In the current study, we sought to provide evidence to support or refute this hypothesis and thus better understand the types of motions that can be regulated by Bpy LG strategy. We focused on addressing two key questions. First, what is the affinity of the Bpy LGs for Ni(II) in the protein context? This value reflects both metal coordination and any conformational rearrangements required for complex formation, providing a thermodynamic measure of switching capacity. And second, does the switch control global domain motions, local conformational changes (e.g., in catalytic loops), or other structural features? To address these questions, we measured the Ni(II) binding affinity of POP variants containing Bpy LGs. In our previous study, several Bpy‐containing POP variants were screened for reversible metal‐dependent switching, and POP_Bpy167/517_ and POP_Bpy169/512_ exhibited the strongest switching behavior. These variants were therefore selected for detailed mechanistic investigation in the present work. While both constructs display similar Ni(II)‐dependent switching activity, they differ in the positioning of the Bpy residues across the β‐propeller and peptidase domains of the POP scaffold. Conformational changes associated with switching were probed using fluorescence resonance energy transfer (FRET) [24, 25] and ^19^F NMR spectroscopy [26, 27, 28]. Finally, experimental data were integrated with molecular dynamics (MD) simulations to construct a structural model of LG induced switching in POP.

## 2 Results and Discussion

### 2.1 Linking Group Metal Binding Affinity

**FIGURE 2:**
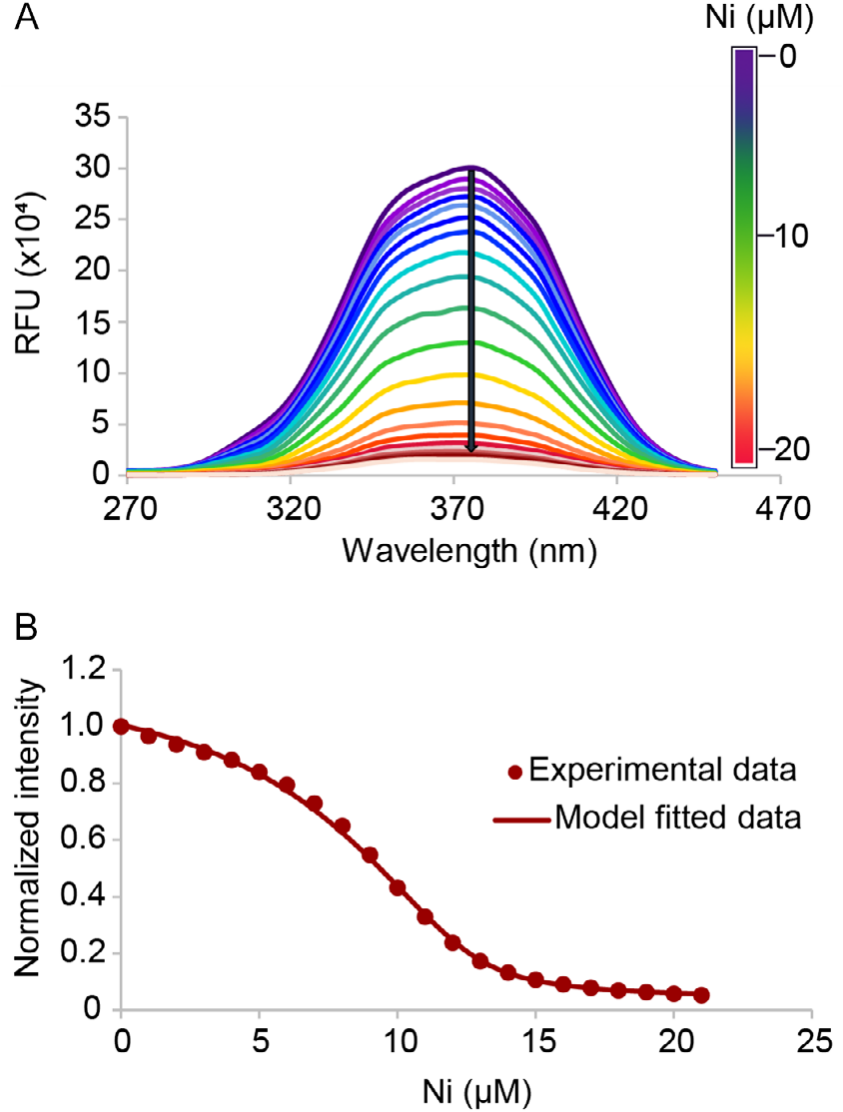
Metal‐binding affinity determination via competition with the fluorescent chelator (Mf2). (A) Fluorescence excitation spectra (λ_em_ = 505 nm) showing a binding competition between POP_Bpy167/517_ and Mf2 upon titration with increasing concentrations of Ni(II), which quenches Mf2 fluorescence emission. Spectra represent the emission intensity at 505 nm (ordinate) as a function of excitation wavelength (abscissa). (B) Binding curve derived from excitation intensity at 372 nm as a function of Ni(II) concentration at room temperature. The apparent K_d_ was determined to be ~ 41 nM by global fitting of data from three independent experiments using a custom Dynafit script.

We initially explored multiple biophysical methods to quantify the Ni(II) binding affinities of POP_Bpy_ variants. Constructs compatible with microscale thermophoresis (MST) [29] and isothermal titration calorimetry (ITC) [30] were prepared; however, both approaches yielded inconclusive results. MST experiments showed no significant changes in thermophoretic signals despite extensive optimization, while ITC was hindered by baseline instability and poor reproducibility (Figure S1). Greater success was achieved using a metal‐binding competition assay with the fluorescent chelator magfura‐2 (Mf2) [31, 32, 33]. The affinity of Mf2 for Ni(II) was first determined by monitoring the decrease in fluorescence at 505 nm due to fluorescence quenching upon complex formation (Figure S2). To determine the apparent binding affinity of POP_Bpy_ variants, Ni(II) was titrated into a solution containing both the POP_Bpy_ variant and Mf2, and the decrease in fluorescence at 505 nm was recorded (Figure 2a). Dissociation constants (K_d_) were extracted by fitting the titration data using DynaFit [34, 35] (Figure 2b). The apparent K_d_ values for POP_Bpy167/517_ and POP_Bpy169/512_ were 41.5 ± 2.6 nM (Figure 2b) and 58.2  ± 4.8 nM (Figure S3), respectively. Single Bpy‐containing controls, POP_Bpy167_ and POP_Bpy517_, exhibited 22.5‐ and 16‐fold weaker binding (apparent K_d_ = 933 and 664 nM, respectively; Figure S4). Similarly, POP_Bpy169_ and POP_Bpy512_ showed 5.7 and 7.8‐fold weaker binding, respectively, compared with the double mutant POP_Bpy169/512_ (Figure S3). Mf2 is not sensitive to weak binding events; thus, it was not suitable for accurately determining the K_d_ of POP_WT_. Instead, this value was measured using a ^19^F NMR titration experiment, yielding an apparent K_d_ of ~ 66 µM, as discussed in a later section and Figure S5.

The apparent K_d_ value for the metal binding to the double (POP_Bpy167/517_) and single (POP_Bpy167_, POP_Bpy517_) Bpy mutants comprises several hidden thermodynamic contributions that are not immediately accessible to experimental measurements. In particular, the experimental measurements cannot separate the contributions from both Ni(II) Bpy binding and the structural changes in protein required for that binding. For this reason, it is of interest to try to shed light on some of these components explicitly using free‐energy calculations based on MD simulations.

**FIGURE 3:**
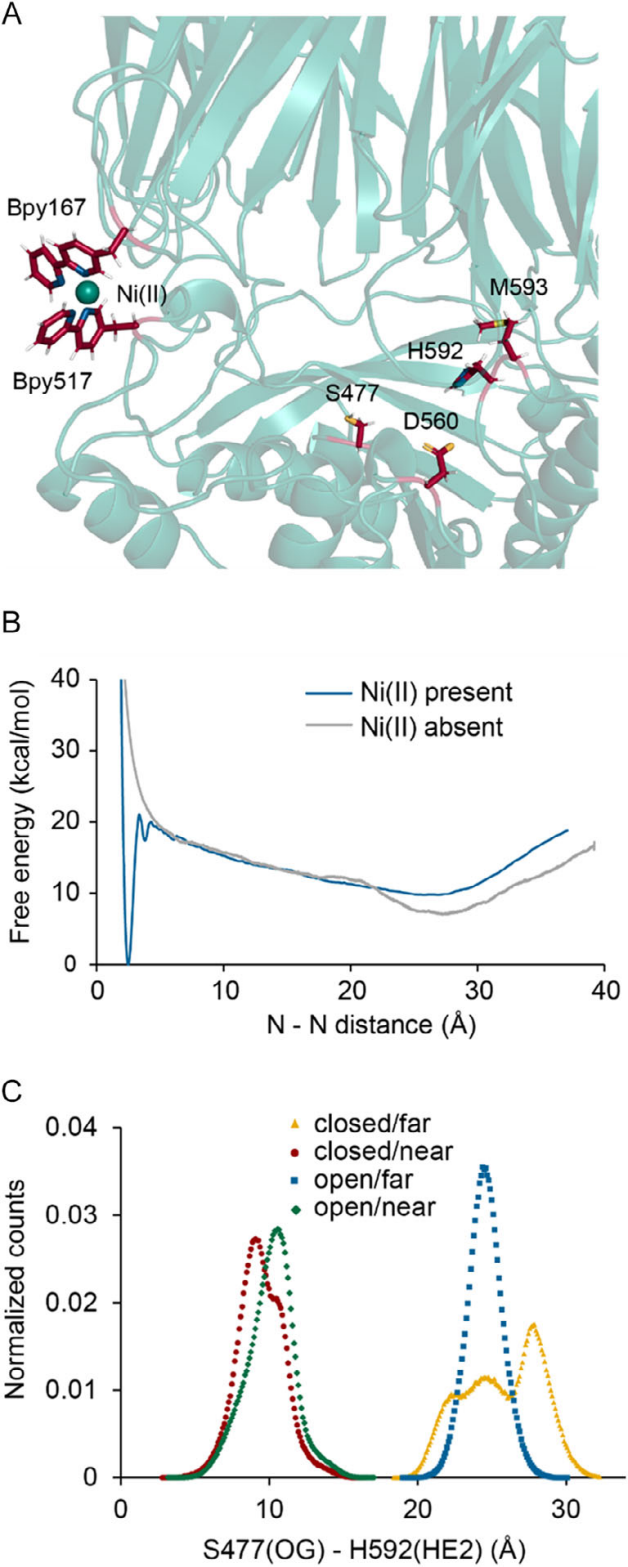
MD simulations of the open/closed conformational change of POP. (A) Ni(II) bound to Bpy167 and Bpy517 in the closed conformation of POP. (B) Potential of mean force (PMF) associated with the open/closed conformational change of POP. The N‐N distance is measured between the centers of mass of the two nitrogen atoms on Bpy167 and the two nitrogen atoms on Bpy517. The PMF in the presence of the Ni(II) was calculated using umbrella sampling from 180 windows simulations (150 ns/window) biased by a 125 kcal/mol/Å^2^ harmonic potential with reference distances ranging from 2 to 37.8 Å with a separation of 0.2 Å. Ni(II) was restrained to Bpy167 by a half‐harmonic potential at 5 Å from the center of the two Bpy nitrogen atoms. In the absence of Ni(II), there are 196 windows (28 ns/window). Postprocessing of the biased data to reconstruct the unbiased free energy landscape was done using the Weighted Histogram Analysis Method (WHAM) [36]. (C) Histograms from simulations of the “near” (red, green) and “distant” (blue, yellow) POP conformations of the catalytic triad in both the open (green and blue) and closed (red and yellow) states. The distance on the *x*‐axis is between the hydroxyl oxygen (OG) of S477 and the imidazole amide hydrogen (HE2) of H592.

As a first step, the potential of mean force (PMF) associated with the open and closed conformation of the double mutant POP_Bpy167/517_ was calculated as a function of the Bpy167‐Bpy517 distance in the absence of Ni(II) using umbrella sampling MD simulations. A second PMF was also calculated with umbrella sampling MD simulations under the condition that one Ni(II) remains associated to Bpy167. The two PMFs are shown in Figure 3B. The backbone root‐mean‐square deviation (RMSD) of the two POP domains during the umbrella sampling simulations is on the order of 1.5 and 2.5 Å, respectively.

At distances larger than 5 Å, the PMFs with and without the metal are qualitatively similar. There is a small difference that may be due to the presence of the metal bound to one Bpy, which remains solvated, while the Bpy without a metal tends to be shielded from the solvent. Both PMFs display a broad free energy minimum corresponding to the open conformation around a distance of 25 Å. However, the two PMFs differ markedly when the distance is smaller than 5 Å, corresponding to the closed conformation. In the absence of the metal, the free energy of the closed conformation is roughly 15 kcal/mol higher than that of the open conformation. In the presence of the metal, however, a deep free energy well stabilizes the closed conformation at a distance of 2.5 Å.

We next sought to reconcile the thermodynamics of binding with the observed behavior. Assuming that the metal binds to the Bpy in the open (o) or closed (c) states, the apparent dissociation constant to the double (POP_Bpy167/517_) Bpy mutants is,

(1) $$K_{\mathrm{app}}^{\left(d\right)}=e^{\text{Δ}G_{M}/\mathrm{kT}}K_{\text{open}}^{\left(d\right)}$$

where Δ*G*_*M*_ represents the free energy of the closed state relative to the open state in the presence of metal and $K_{\text{open}}^{\left(d\right)}$ is the dissociation constant to a single Bpy (assumed to be the same here for the sake of simplicity). Similarly, one could also express the apparent dissociation constant $K_{\mathrm{app}}^{\left(d\right)}$ as $e^{\text{Δ}G_{0}/kT}K_{\text{closed}}^{\left(d\right)}$, where $\text{Δ}G_{0}$ represents the free energy of the closed state relative to the open state in the absence of metal, and $K_{\text{closed}}^{\left(d\right)}$ is the dissociation constant to a pre‐arranged double (POP_Bpy167/517_) Bpy mutant in the closed POP conformation. The free energies Δ*G*_0_ and Δ*G*_M_ are calculated from the PMF shown in Figure 3B by integrating the Boltzmann factor, $\exp\;(-W_{s}(x)/k_{B}T)$, over the region of *x* corresponding to the basin of the state *s*,

(2) $$\text{Δ}G_{s}=-k_{B}T\;\ln\;\frac{\int_{0}^{d}e^{-W_{s}(x)/k_{B}T}dx}{\int_{d}^{\infty }e^{-W_{s}(x)/k_{B}T}dx}$$

where *s* means with or without the metal (subscript M or 0). A distance *d* of 4 Å was chosen because it is the maximum distance for the deep and narrow well where the metal is doubly coordinated by Bpy167 and Bpy517 according to the PMF (Figure 3B). Values of + 16.5 and −7.6 kcal/mol were obtained for Δ*G*_0_ and Δ*G*_M_, respectively. Based on the experimental binding data, the dissociation constant to Bpy167 is 933 nM. This value is a reasonable estimate for $K_{\text{open}}^{\left(d\right)}$. Accordingly, the apparent dissociation constant of POP_Bpy167/517_ should be around 0.0026 nM, which is smaller than the measured value of 41 nM. To match the experimental observation, ΔG_M_ should be on the order of −2 or −3 kcal/mol rather than the calculated value of −7.6 kcal/mol. Even though the direction of the shift caused by the metal bridge is qualitatively correct, there is a considerable quantitative discrepancy. Here, it is important to recall that the Bpy‐metal force field model was adjusted to yield the correct binding of Ni(II) to one and two Bpy ligands (Figure S14). The origin of the discrepancy is likely to be the lack of sampling of the open POP conformation in the umbrella sampling calculations, which would shift the estimated free energy Δ*G*_M_ to a smaller value.

While the open/closed state of POP can be controlled by the formation of the Bpy metal bridge, the simulations revealed that the loop containing H592 also undergoes significant conformational changes independent of the Bpy_2_M linkage. H592, S477, and D560 comprise the catalytic triad [37] of POP, and two distinct conformations of the H592 loop are observed. The conformation in which H592 approaches S477 and D560 is similar to the structure of inhibitor bound structures, whereas the conformation in which the residues of the triad are distant from one another is presumably not catalytically competent. These “near” and “distant” conformations appear to be accessible for both the open and closed state of POP (Figure 3C). Although these do not interconvert on the timescale of the simulations while POP is in the open or the closed state, the conformational heterogeneity of the H592 loop in the open and close states shows that activity switching driven by metal binding cannot be attributed to simple open/closed motions alone.

### 2.2 Conformational Changes Upon Metal Binding

**FIGURE 4:**
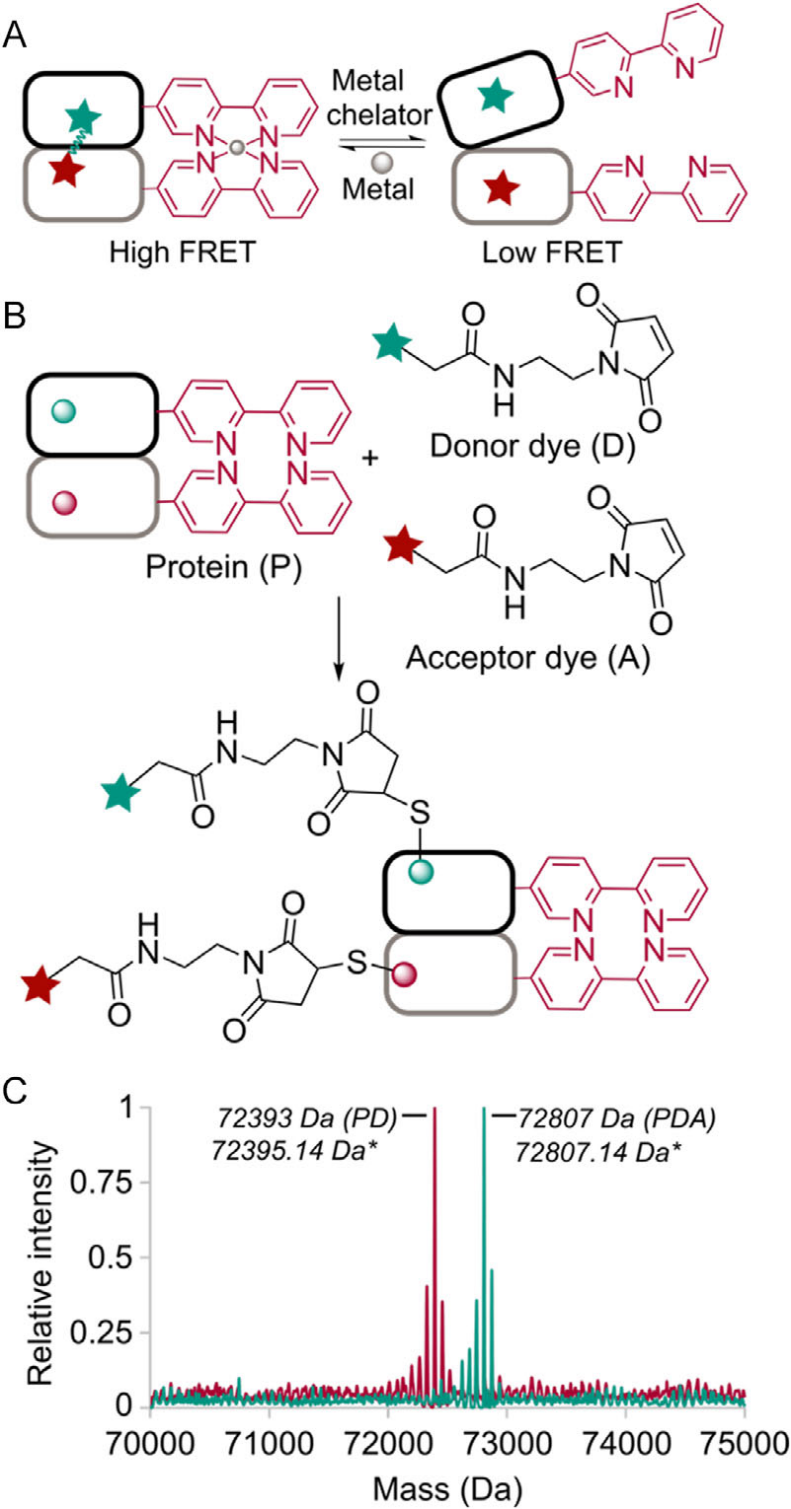
FRET strategy for monitoring POP conformational dynamics. (A) High FRET state (in the presence of metal) and low FRET state (in the absence of metal). (B) Bioconjugation strategy for FRET dye installation. (C) Deconvoluted ESI‐MS spectra of intact protein showing PD and PDA products, respectively (*Theoretical mass).

We next sought to experimentally probe the nature of conformational changes that occur in POP variants containing Bpy LGs upon metal binding. We initially explored ^13^C‐methyl‐TROSY NMR, aiming to capture domain motions similar to those reported by Giralt et al. [38] for porcine POP in the presence of inhibitors, but low protein yields (<1 mg/L) of the Bpy variants in methionine‐auxotrophic strains limited this approach. We next examined whether fluorescence resonance energy transfer (FRET) could be used to determine whether such motions were occurring. The two possible FRET states that we hypothesized for Bpy‐containing POP switches are the low FRET state (open/metal unbound) and high FRET state (closed/metal bound) (Figure 4A). To bioconjugate the FRET dye pair (donor and acceptor dyes), we selected 256 and 513 sites out of various predicted sites based on previous MD simulation data (Figure S6 and Table S1). The selected FRET sites are far from the Bpy switching sites to avoid interfering with the reversible switching. These two sites also provide a large difference in Cβ distances between the two states to differentiate via FRET (open 35.3 Å vs. closed 23.1 Å). Based on the predicted Cβ distances between sites 256 and 513 in our earlier study [12], the maleimide derivative of EDANS (donor dye) and DABCYL (acceptor dye, dark quencher) FRET dye pair was selected, whose literature‐reported Förster distance (R) is 33 Å [39, 40, 41]. The sites 256 and 513 were mutated to cysteine to bioconjugate the FRET dye pair through cysteine maleimide cross‐linking.

**FIGURE 5:**
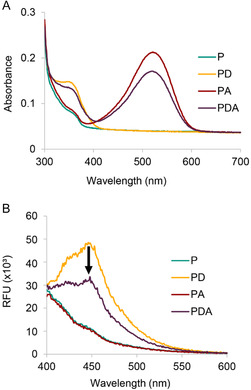
Spectroscopic characterization of POP‐dye conjugates. (A) UV–vis absorption spectra of POP‐dye conjugates (50 µM), showing characteristic absorption maxima at 350 nm for the donor dye (PD), 520 nm for the acceptor dye (PA), and both peaks in the dual‐labeled conjugate (PDA). (B) Fluorescence emission spectra of the conjugates (1 µM) upon excitation at the donor's maximum (λ_ex_ = 341 nm). Quenching of the donor emission (λ_em_ = 448 nm) is observed in the PDA conjugate, where both donor and acceptor dyes are present, as indicated by the black arrow (yellow to purple trace). The acceptor dye, DABCYL, acts as a dark quencher for the donor dye, EDANS, confirming energy transfer within the dual‐labeled system.

To ensure maximum FRET efficiency, we first optimized the bioconjugation of the donor and acceptor dyes to the POP_WT_ (Figure 4B). A 1:2 protein‐to‐dye ratio of either dye provided optimal labeling efficiency, as confirmed by intact protein ESI‐MS (Figures S7 and S8). We next wondered if site‐selective labeling might be possible based on the relative reactivity of the two cysteine residues. Simultaneous labeling using a 1:2:2 ratio of protein:donor:acceptor (P:D:A) provided similar amounts of singly labeled PD and PA products, with only a trace amount of the doubly labeled PDA product (Figure S9), indicating that one site reacts significantly faster than the other. We therefore adopted a sequential labeling strategy involving initial reaction with the donor dye to form PD, followed by reaction of PD with 10 equivalents of the acceptor dye to give PDA in high yield (Figure S9). Under these optimized conditions, both PD and PDA were obtained with > 99% labeling efficiency for the POP_Bpy167/517_ variant (Figure 4C). Successful bioconjugation was further confirmed by UV‐Vis spectroscopy, which showed the characteristic λ_max_ of each dye in the singly (PD or PA) and doubly (PDA) labeled products (Figure 5A). To ensure that labeling did not interfere with metal‐dependent switching, a previously established steady‐state kinetics assay [12] was performed in the presence and absence of metal (Figure 1C). The results demonstrated that the POP_Bpy167/517_ variant retained its switching activity after dye conjugation (Figure S10).

To evaluate conformational changes via FRET, we first measured fluorescence emission spectra upon excitation at 341 nm (donor λ_ex_). The PDA‐labeled POP_Bpy167/517_ variant showed quenched donor emission compared to the singly labeled PD control, indicating that FRET was occurring (Figure 5B ). We next quantified FRET efficiency (E_FRET_) in the presence and absence of Ni(II). Based on theoretical calculations, we expected a significant change in E_FRET_ upon metal binding, specifically, an increase from ~0.4 in the open (metal‐free) state to ~ 0.9 in the closed (metal‐bound) state. However, no significant difference in E_FRET_ was observed between the two states; both remained around 0.4 (Table S2, Supporting Information). This suggested that the distance between residues 256 and 513 did not change appreciably upon switching.

**FIGURE 6:**
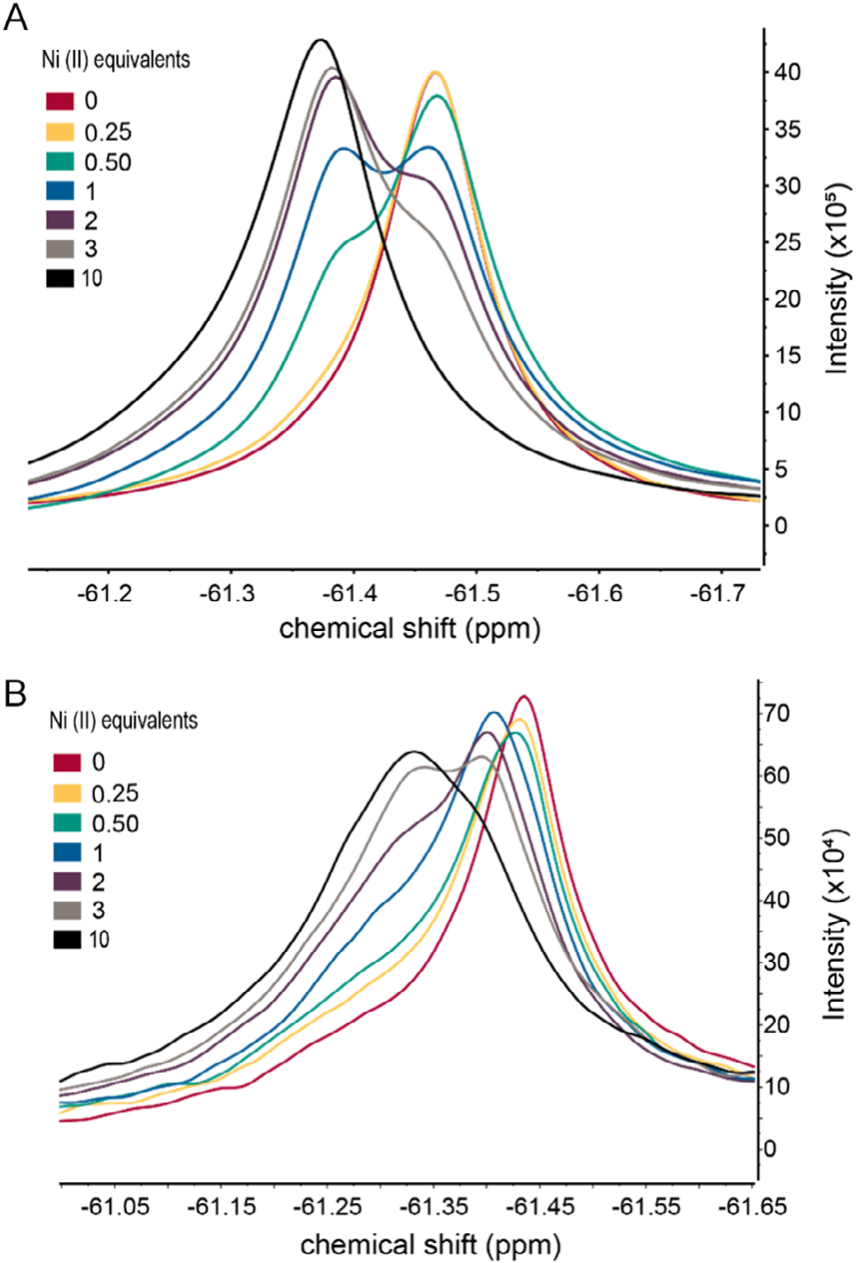
^19^F NMR spectra of POP variants at varying equivalents of Ni(II). (A) ^19^F NMR spectra of POP_WT_ showing that in the absence of Bpy LGs, no high‐affinity binding is observed. Instead, a gradual shift in population toward a new state is observed with increasing Ni(II), consistent with nonspecific binding. The dissociation constant (K_d_) for this weaker interaction is approximately 66 µM (Figure S5). (B) ^19^F NMR spectra of POP_Bpy167/517_ variant under identical conditions. At 1 equivalent of Ni(II), a new peak appears, corresponding to a distinct population arising from a high‐affinity binding event between the Bpy and Ni(II). At higher Ni(II) concentrations, a third peak emerges, attributed to a secondary, nonspecific binding event with weaker affinity.

Analysis of the umbrella sampling simulations described above revealed that the correlation between sites 256 and 513 and the open‐close motion of the POP_Bpy167/517_ was weak. This was evident by the negative slope of the least squares fit between the distance of the N‐N centers of mass of the two Bpy groups and the Cβ distance between sites 256 and 513, predicting minimal involvement of these sites in the conformational dynamics (Figure S11). These later simulations also indicated that sites 161 and 520 correlate better with open‐close motion in POP_Bpy_ variants, as the slope defined above is positive for these new sites. FRET dyes were conjugated to sites 161 and 520 in POP_Bpy167/517_ and POP_Bpy169/512_ using labeling conditions described above (Figure S12). However, despite these efforts, the FRET efficiency did not show significant changes between the metal‐free and metal‐bound states in both switches. This lack of change in E_FRET_ suggests that, although sites 161 and 520 are more closely tied to the open‐close motion in simulations, metal binding did not induce a noticeable conformational shift detectable by FRET (Table S2).

The absence of significant changes in FRET efficiency upon metal binding across multiple FRET sites and Bpy variants suggests that such large domain motions are not the primary mechanism regulating activity. We therefore examined whether more localized structural changes, such as loop motions near the active site observed in the MD simulations noted above, could play a role in modulating activity upon metal coordination. These smaller‐scale rearrangements, while not detectable by FRET, may be sufficient to alter substrate access or catalytic geometry and thus inhibit enzymatic function in the metal‐bound state. Because ^19^F NMR spectroscopy has been widely used to examine such subtle conformational changes in proteins [26, 27, 28, 42], we introduced M593C into POP to enable site‐specific incorporation of a fluorinated probe, 2‐bromo‐N‐(4‐(trifluoromethyl)phenyl)acetamide (BTFMA) [42], adjacent to the catalytic H592 residue. BTFMA was attached via cysteine–acetamide crosslinking, achieving > 99% labeling efficiency (Figure S13).

First, we performed a Ni(II) titration with POP_WT_ containing the BTFMA probe at site 593 (Figure 6A). The shift in population observed was due to weak, nonspecific binding, with a K_d_ of ~ 66 µM (Figure S5). Importantly, this binding event does not interfere with enzymatic activity, as shown in our previous study [12], where we demonstrated that POP_WT_ remains active even in the presence of excess Ni(II). This suggests that the nonspecific binding is not a major driver of switching behavior, as the enzyme remains largely functional at these Ni(II) concentrations. In contrast, titration of the POP_Bpy167/517_ variant revealed a new population at one equivalent of Ni(II), which we attribute to the stronger binding event at the Bpy sites, with a K_d_ of ~ 41 nM (as noted above), resulting in activity inhibition (Figure 6B). When Ni(II) exceeds one equivalent, another population emerges, resembling the nonspecific binding population observed in POP_WT_. This suggests that in the Bpy‐containing protein, there are two binding events: one with strong affinity for Bpy and another with weaker nonspecific binding. These findings suggest that the population shift at low Ni(II) equivalents in the Bpy variant results from local loop rearrangements associated with Bpy binding, which disrupt enzymatic activity, highlighting the role of local conformational changes in regulating the protein's function.

## 3 Conclusion

In this study, we examined the mechanism by which genetically encoded Bpy linking groups regulate the activity of *Pyrococcus furiosus* prolyl oligopeptidase (*Pfu* POP) in response to Ni(II). Ni(II) binding was found to stabilize a catalytically inhibited state with nanomolar affinity. PMF calculations provided qualitative agreement with the corresponding Gibbs free energy change (ΔG), validating our computational thermodynamic model for predicting switching behavior. This model provides a means to examine the utility of the Bpy linking group strategy in other proteins; before performing experiments, one can computationally evaluate the feasibility of switching by estimating the expected energetics in silico, thereby streamlining experimental design and reducing development time.

FRET and ^19^F NMR experiments revealed that activity modulation is not mediated by large‐scale domain motion but instead by local rearrangements near the catalytic triad. This observation aligns with our molecular dynamics simulations, which show that the loop containing H592 undergoes significant conformational changes that lead to a catalytically inhibited state. The lack of global domain motion also explains why microscale thermophoresis (MST) experiments showed minimal changes in thermophoretic signal, as MST primarily detects larger conformational shifts [43]. Together, these results establish that local structural modulation is sufficient to inhibit catalysis, broadening the applicability of this switching strategy to proteins that lack large‐scale domain motions.

## Supporting Information

Additional supporting information can be found online in the Supporting Information section. Additional supporting information, including supplementary figures, materials, and methods, can be found online in the Supporting Information section. The authors have cited additional references within the Supporting Information [44, 45, 46, 47, 48, 49, 50, 51, 52, 53, 54, 55].

## Funding

This work was supported by the Army Research Laboratory (W911NF‐18‐1‐0200) and the National Institutes of Health (T32 GM131994).

## Conflicts of Interest

The authors declare no conflicts of interest.

## Supporting information

Supplementary Material

## Data Availability

The data that supports the findings of this study are available in the supplementary material of this article.
