# Supplementary material for: Mechanistic Insight into Conformational Control of Enzyme Activity by Genetically Encoded Metal‐Responsive Switches
Source: Chembiochem. 2026 Apr 24;27(8):e70349. doi: 10.1002/cbic.70349 (PMC13109687; doi:10.1002/cbic.70349)
Supplement: Supplementary file 1 — Supplementary Material [file CBIC-27-e70349-s001.pdf]

Mechanistic Insight into Conformational Control of Enzyme Activity by Genetically-Encoded Metal-Responsive Switches

Payal[1], Jonathan Thirman[2], Katherine A Edmonds[1], Sandip Mishra[1], Nathan Blackwell[1],Yasmine S Zubi[1], Benoît Roux[2,*], Jared C. Lewis[1,*]

[1]Department of Chemistry, Indiana University, Bloomington, Indiana 47405, United States
[2] Department of Biochemistry and Molecular Biology, University of Chicago, Chicago, IL, 60637, USA
* jcl3@iu.edu and roux@uchicago.edu

Table of Contents

I. Supplementary figures and tables 2

II. Material and methods 11

A) Materials 11

B) Methods 12

C) Software 12

III. Enzyme preparation 20

A) Cloning of POP variants 20

B) Protein expression and lysis 22

C) Protein purification 22

IV. Biophysical techniques 23

V. Synthetic methods and materials 25

VI. Computational methods 28

VII. References 33

I. Supplementary figures and tables

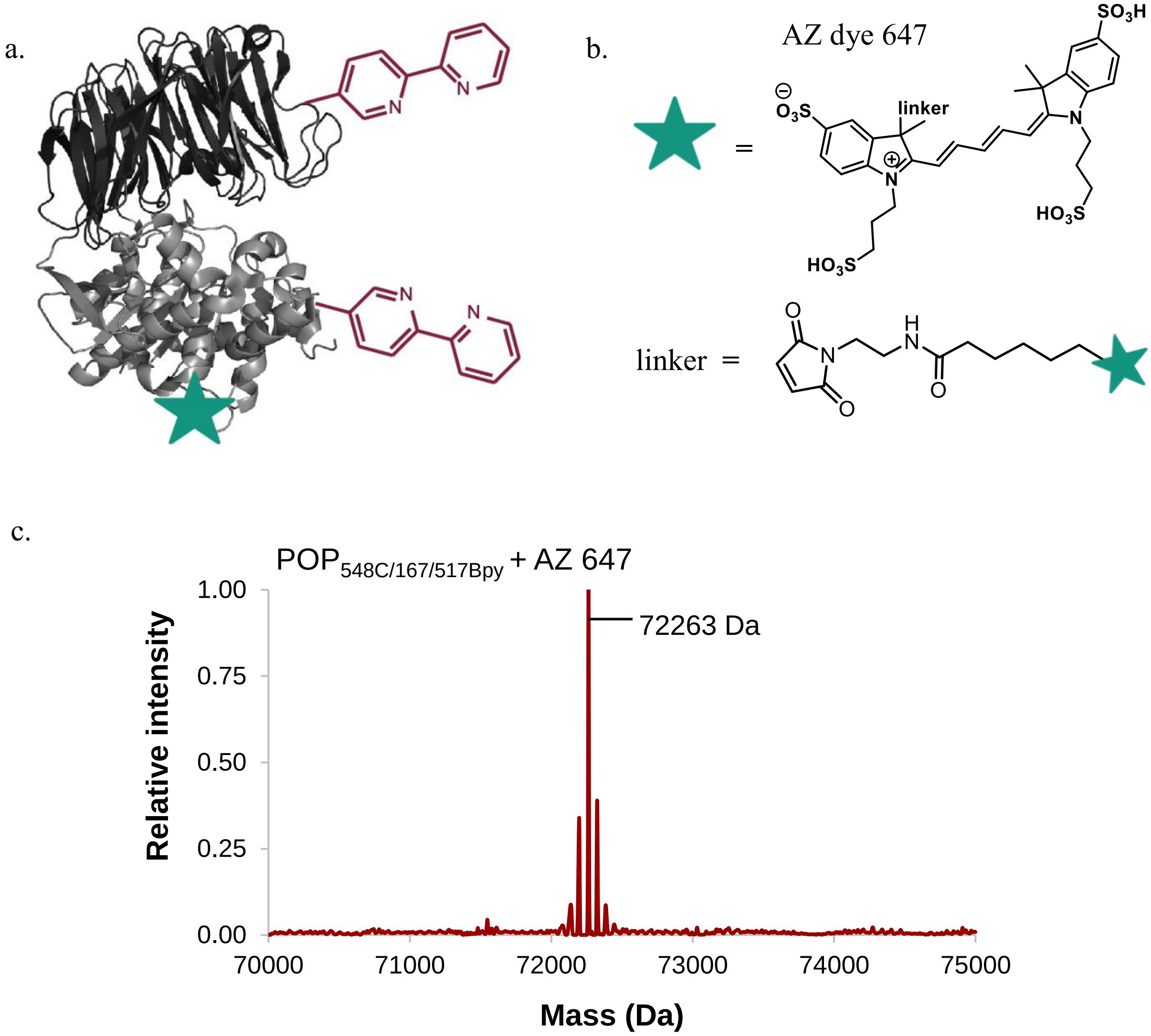

Figure S1. Bioconjugation of dye for MST. **a)** Cartoon representation of the Bpy POP variant showing the site 548C where the labeling of AZ dye 647 was carried out. **b)** Chemical structure of the maleimide derivative of AZ dye 647. **c)** Deconvoluted Intact protein ESI-MS data showing 100 % labeling of the POP variant. The theoretical mass of apo $POP_{548C/Bpy167/517}$ (Δ His tag) is 71275.16 Da, AZ 647 dye is 994.25 Da and dye-labeled protein is 72269.41 Da.

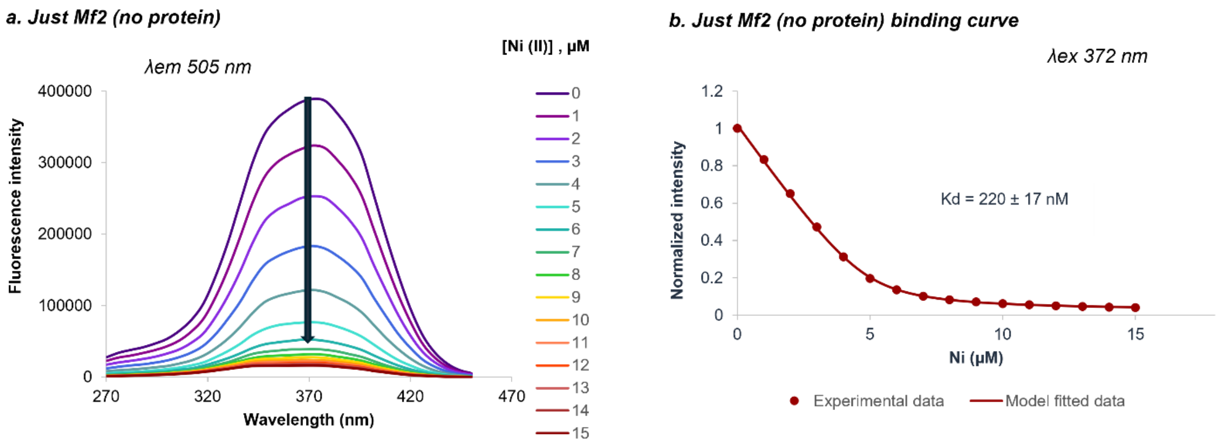

Figure S2. Determination of the binding affinity of magfura2 (Mf2) for Ni^{2+}. **a)** Fluorescence excitation spectra (at λem = 505 nm) of Mf2 titrated with increasing concentration of Ni^{2+}. The binding of Ni^{2+} quenches Mf2 fluorescence. The spectra represent the emission intensity at 505 nm (ordinate) as a function of excitation wavelength (abscissa). **b)** The plot represents the fit of the fluorescence excitation intensity of Mf2 at 372 nm as a function of metal ion concentration at room temperature. K_d values are determined by using a custom Dynafit script (Script#1, SI) by fitting the experimental data from a single measurement.

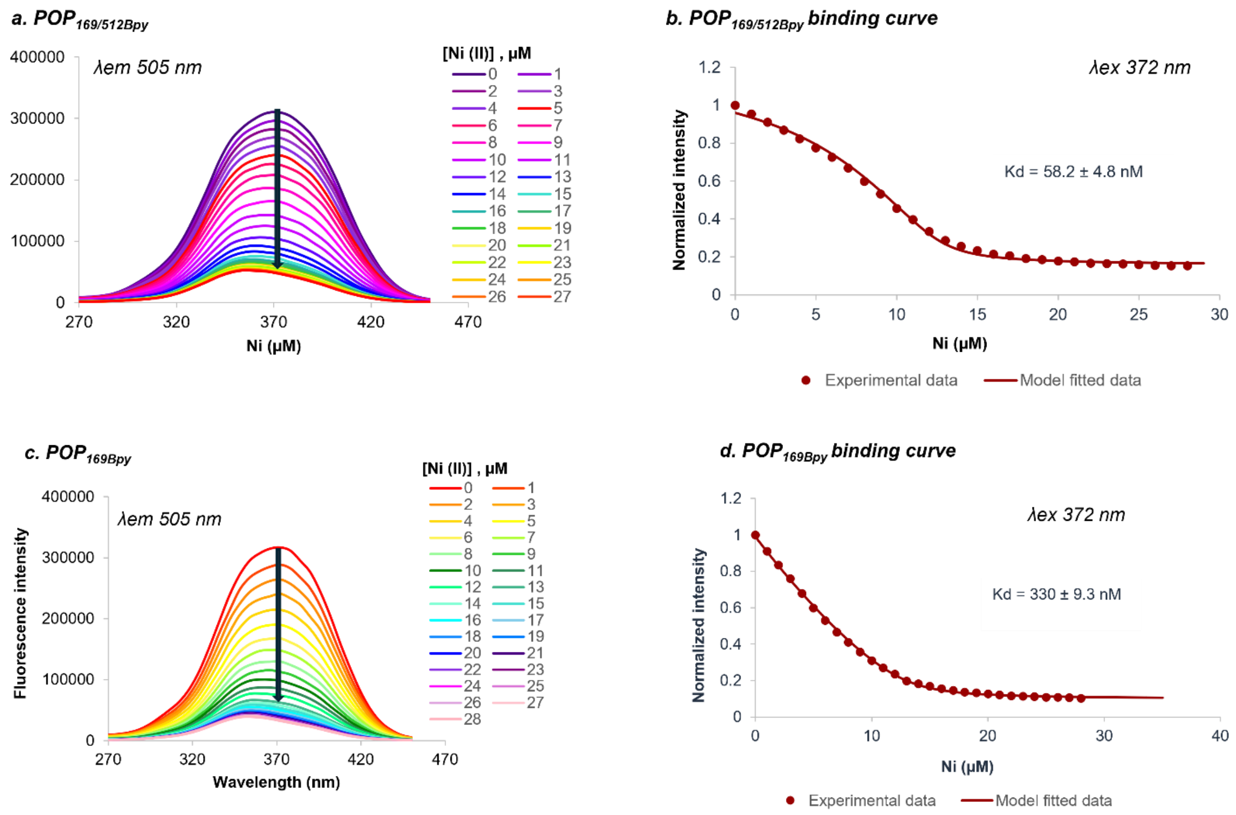

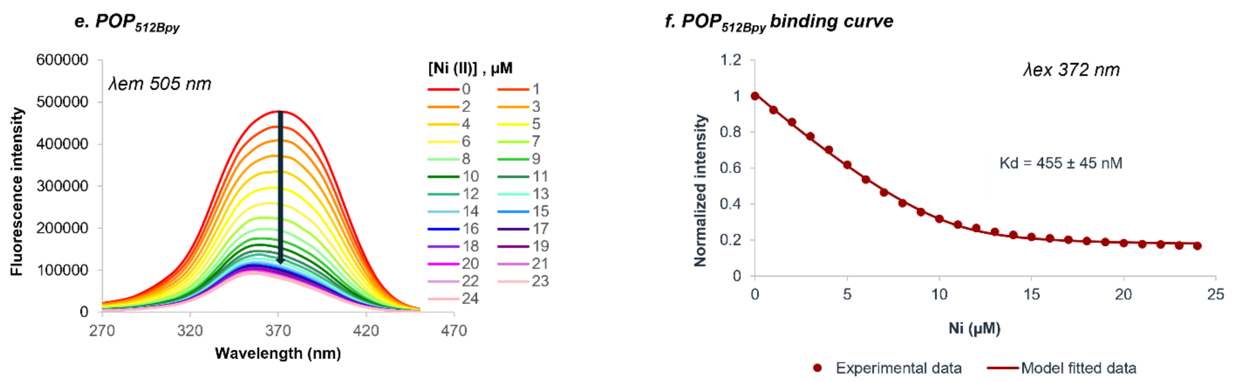

Figure S3. Determination of binding affinities of double Bpy-containing variant ($POP_{Bpy169/512}$) and single Bpy-containing variants (POP_{Bpy169} and POP_{Bpy512}) for Ni^{2+}**. a), c), and e)** are fluorescence excitation spectra (at λem = 505 nm) of a competition experiment between $POP_{Bpy169/512}$ or POP_{Bpy169} or POP_{Bpy512} and Mf2 titrated with increasing concentrations of Ni^{2+}. The binding of Ni^{2+} quenches Mf2 fluorescence. The spectra represent the emission intensity at 505 nm (ordinate) as a function of excitation wavelength (abscissa). **b), d), and f)** are the fit of the fluorescence excitation intensity at 372 nm as a function of metal ion concentration at room temperature for $POP_{Bpy169/512}$ and POP_{Bpy169} and $POP_{Bpy512,}$ respectively. K_d values are determined by using a custom Dynafit script (Script #4, #5, and #6, SI) by global fitting of experimental data from three independent measurements in the case of $POP_{Bpy169/512}$ and $POP_{Bpy169,}$ whereas in the case of $POP_{Bpy512,}$ the K_d value is from a single measurement.

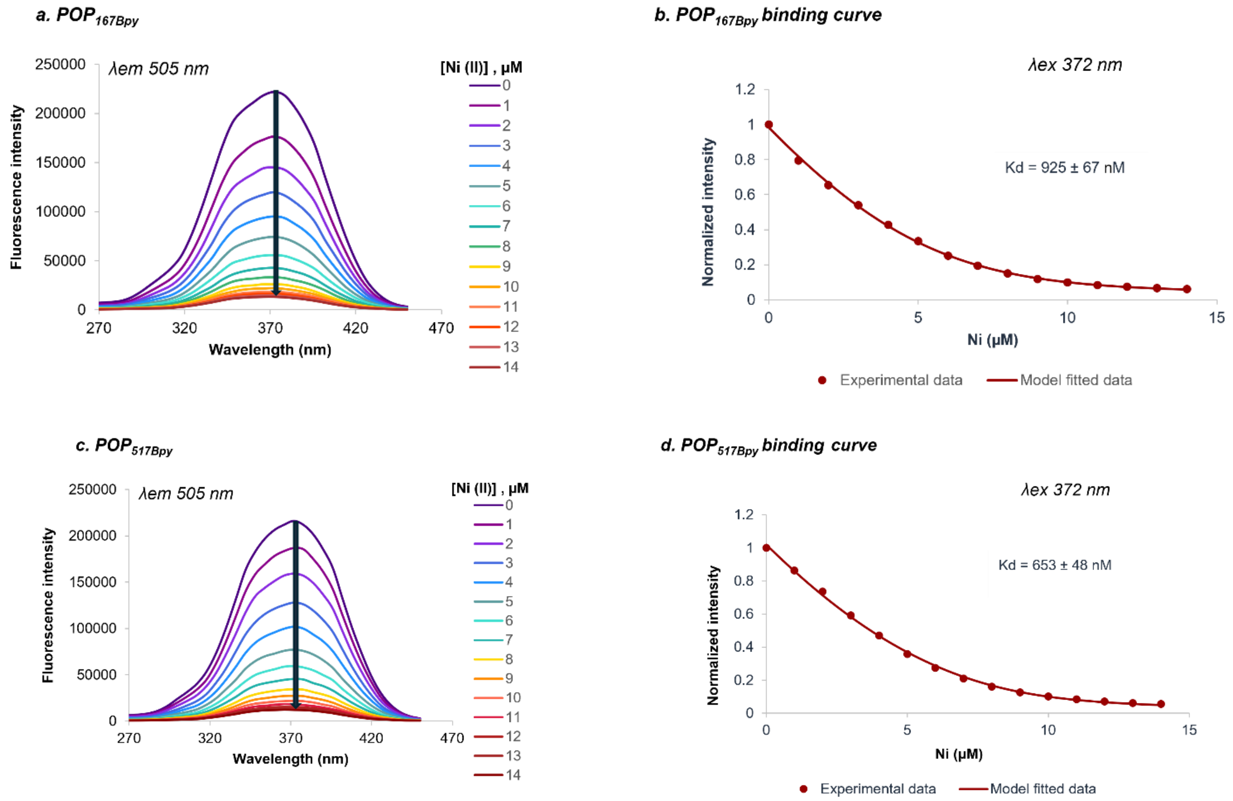

Figure S4. Determination of binding affinities of single Bpy-containing variants (POP_{Bpy167} and POP_{Bpy517}) for Ni^{2+}**. a) and c)** are fluorescence excitation spectra (at λem = 505 nm) of a competition experiment between POP_{Bpy167} or POP_{Bpy517} and Mf2 titrated with increasing concentrations of Ni^{2+}. The binding of Ni^{2+} quenches Mf2 fluorescence. The spectra represent the emission intensity at 505 nm (ordinate) as a function of excitation wavelength (abscissa). **b)**

and d) are the fit of the fluorescence excitation intensity at 372 nm as a function of metal ion concentration at room temperature for POP_{Bpy167} and POP_{Bpy517}, respectively. K_d values in both cases are determined by using a custom Dynafit script (Script #7 and #8, SI) by fitting experimental data from a single measurement.

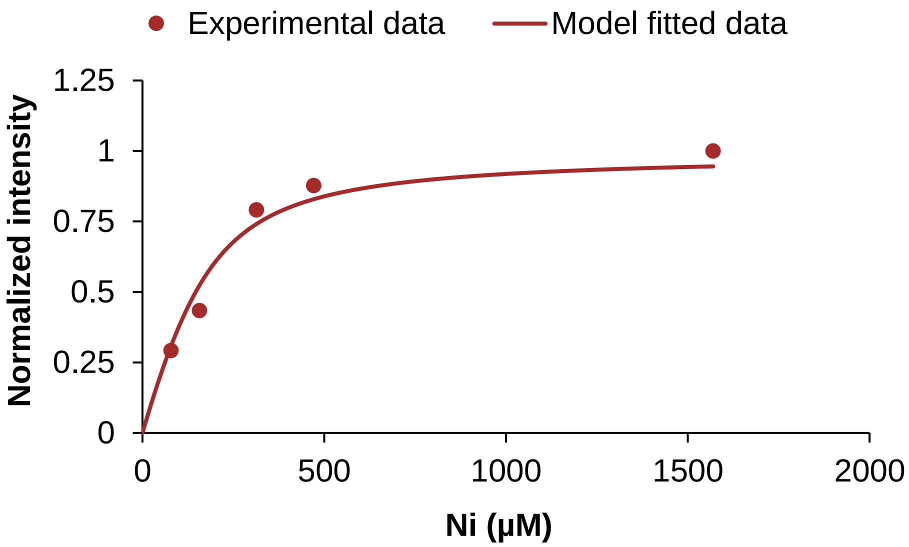

Figure S5. Determination of the weaker binding affinity of POP_{WT} to Ni(II) by ^{19}F NMR spectroscopy. The apparent K_d value was determined to be 66 μM by fitting the ^{19}F NMR titration data using Dynafit (script # 3).

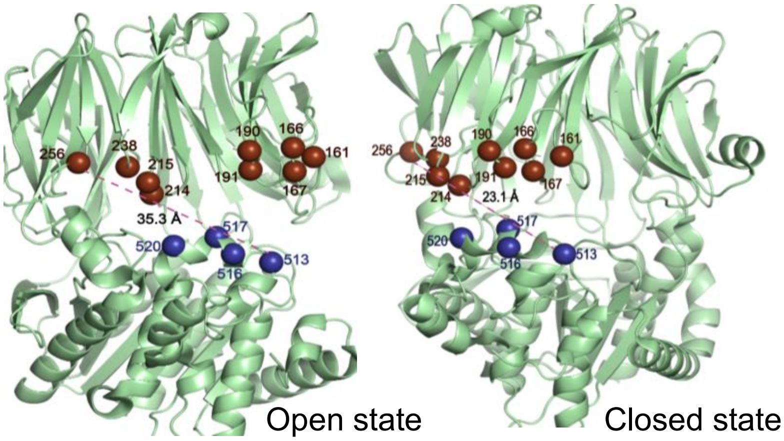

Figure S6. Various site pairs predicted for the installation of FRET dyes from previous MD simulation data.

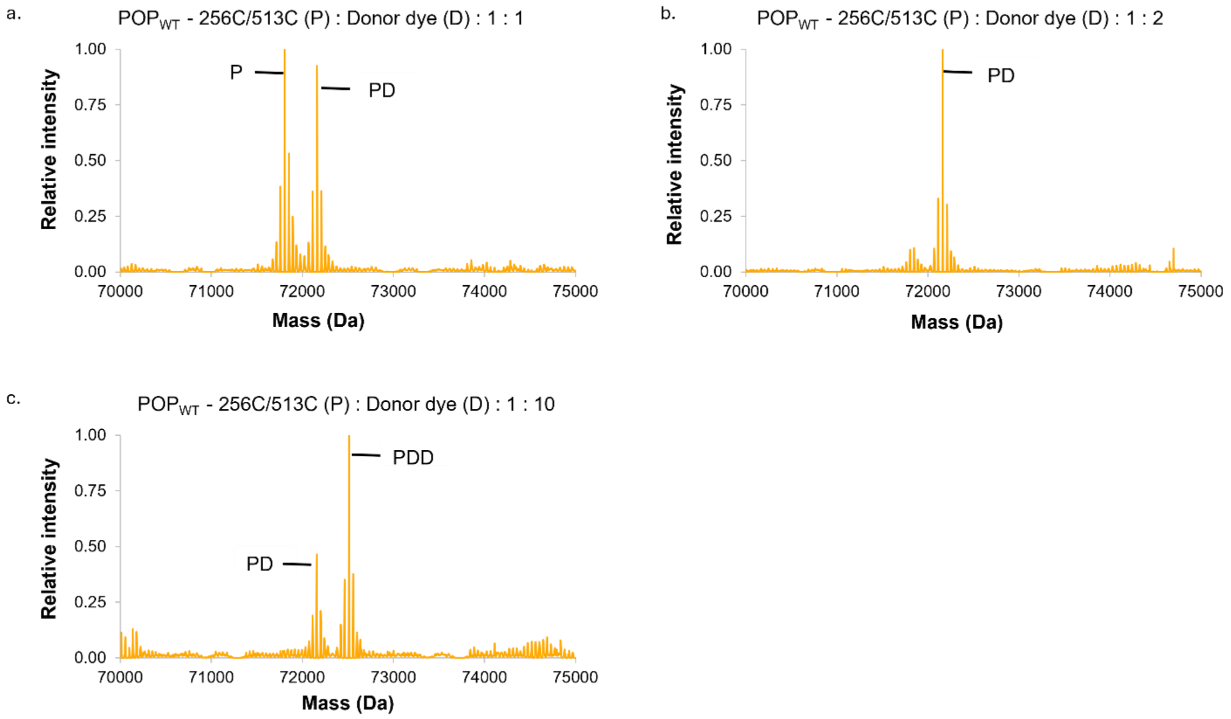

Figure S7. Bioconjugation optimization for the PD product formation in POP_{WT} - 256C/513C (P) variant. Out of the three ratios of P:D, the 1:2 ratio is the optimal ratio with a reaction completed within 10 minutes. The theoretical masses of P, PD, and PDD are 71803, 72158, and 72513 Da, respectively. The experimental masses of P, PD, and PDD from intact protein ESI-MS are 71806, 72161, and 72513 Da, respectively.

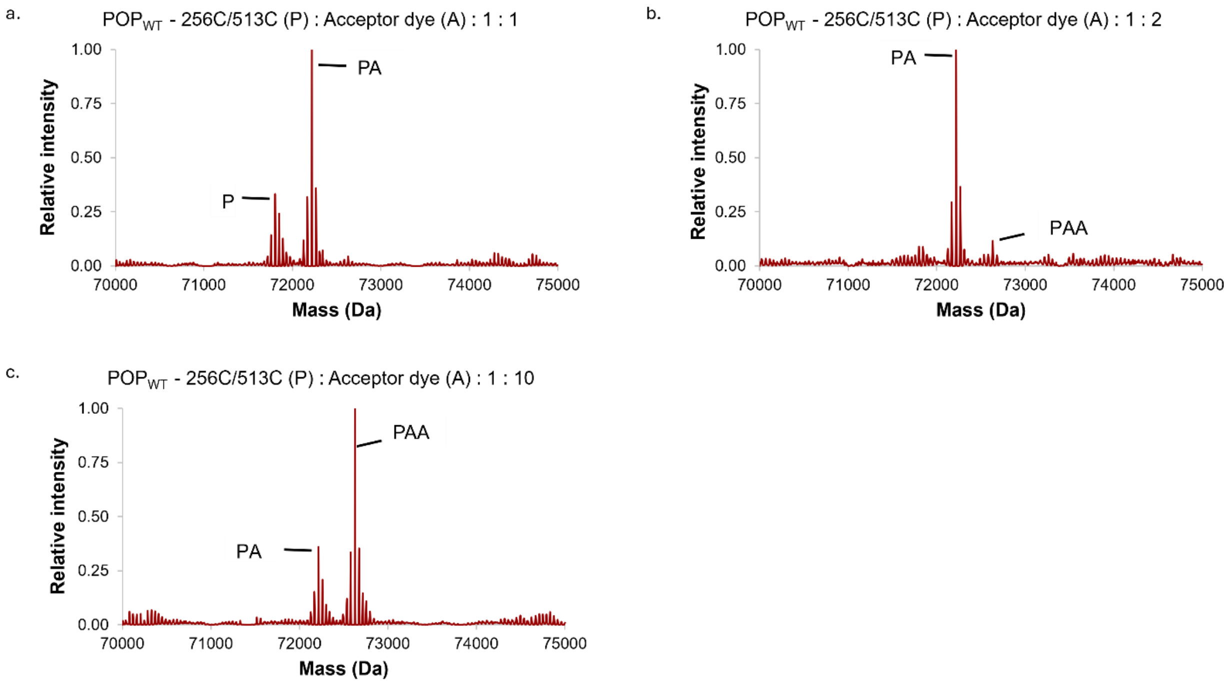

Figure S8. Bioconjugation optimization for the PA product formation in POP_{WT} - 256C/513C (P) variant. Out of the three ratios of P:A, the 1:2 ratio is the optimal ratio with a reaction completed within 10 minutes. The theoretical

masses of P, PA, and PAA are 71803, 72215, and 72627 Da, respectively. The experimental masses of P, PA, and PAA from intact protein ESI-MS are 71805, 72219, and 72628 Da, respectively.

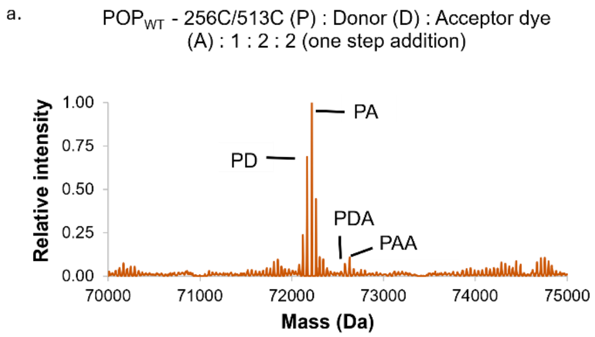

Figure S9. Bioconjugation optimization for the PDA product formation in POP_{WT} - 256C/513C (P) variant. **a)** shows the intact protein ESI-MS of the control, where both the dyes (D and A) were added simultaneously to the protein in a P:D: A:1:2:2 ratio. **b)** shows the intact protein ESI-MS where the reaction was done stepwise. First, 2 equivalents of D were added to P to make PD. After 10 minutes, 10 equivalents of A were added to make PDA. The theoretical masses of P, PD, PDD, PA, PAA, and PDA are 71803, 72158, 72513, 72215, 72627, and 72570 Da, respectively. The experimental masses of P, PD, PDD, PA, PAA, and PDA from intact protein ESI-MS are n/a, 72162, n/a, 72219, 72628, and 72574 Da, respectively.

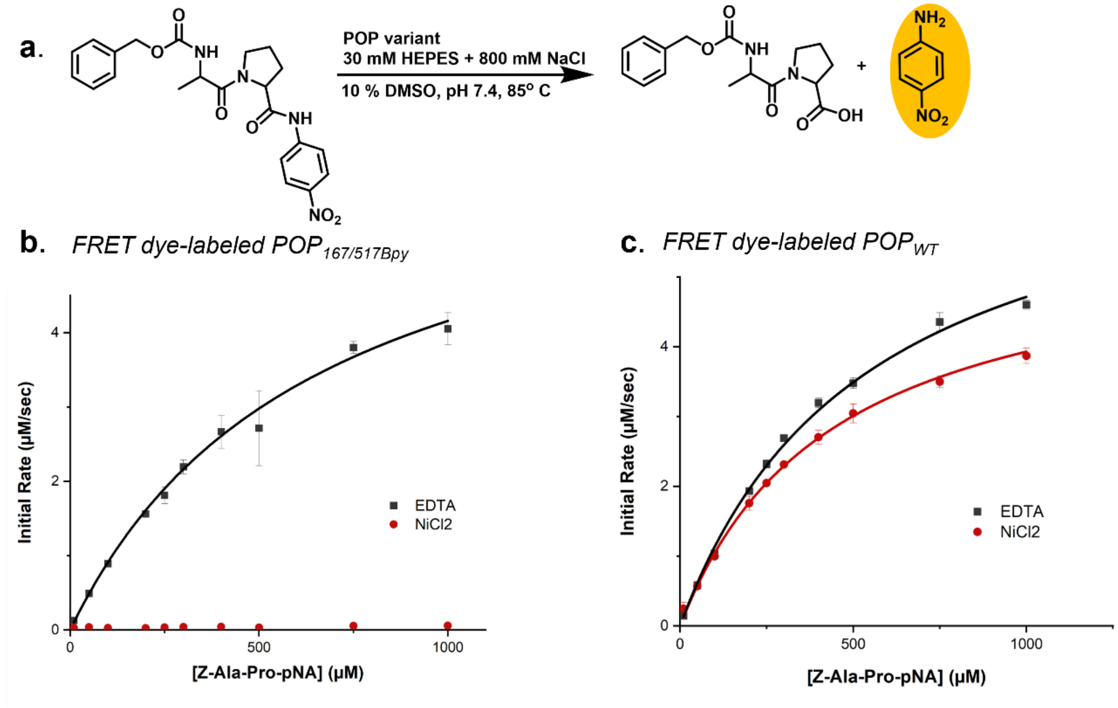

Figure S10. Steady-state kinetics of FRET dye pair conjugated POP variants. **a)** Reaction scheme for native hydrolase activity of POP enzyme towards Z-Ala-Pro-pNA substrate. The product para- nitroaniline (pNA) is a chromogenic (yellow color) which shows maximum absorption at 410 nm. **b) & c)** Steady-state kinetic assays (reference) performed in the presence of either 1 mM EDTA or 5 μM $NiCl_2$ for FRET dye bioconjugated products (PDA) of both $POP_{167/517Bpy}$ and $POP_{WT,}$ respectively. Initial rates (μM/sec) are plotted versus substrate concentration (μM), and data are fitted with the Michaelis-Menten equation.

A.

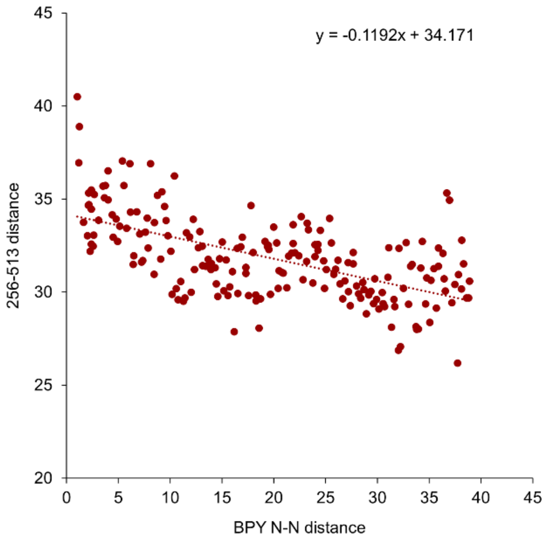

B.

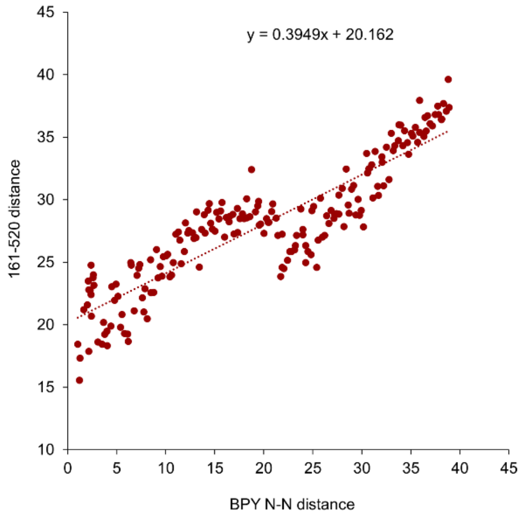

C.

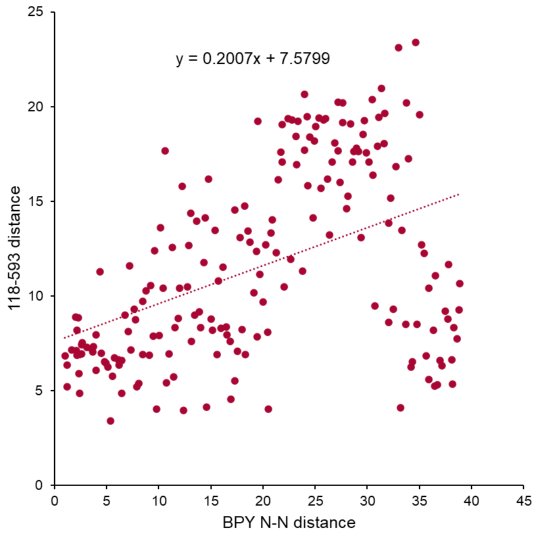

Figure S11. Correlation between the Bpy167-Bpy517 distance and the Cβ-Cβ distance for three residue pairs: 256-513 (A), 161-520 (B), and 118-593 (C). One representative frame was selected from each umbrella sampling window and plotted according to its Bpy167-Bpy517 and Cβ-Cβ distances. A least-squares best-fit line is shown for each dataset. The Cβ-Cβ distance of the 161-520 pair exhibits the strongest correlation with the open-close motion, as reflected by the Bpy167-Bpy517 distance. The Bpy167-Bpy517 distance is defined as the distance between the centers of the two nitrogen atoms of one Bpy group and those of the other.

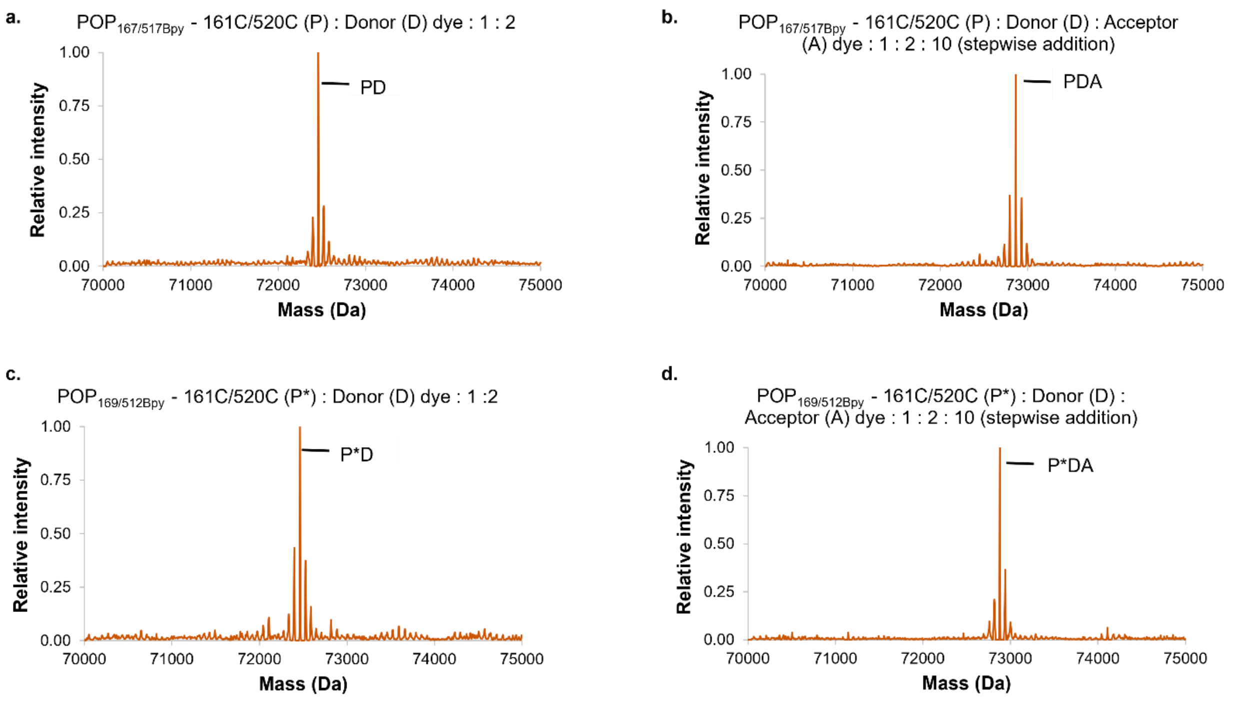

Figure S12. Bioconjugation of FRET dyes at site pair 161 and 520 in $POP_{167/517Bpy}$-161C/520C(P) and $POP_{169/512Bpy}$-161C/520C(P*) variants. **a)** and **b)** show the intact protein ESI-MS for PD and PDA product formation for $POP_{167/517Bpy}$-161C/520C(P) variant. **c) and d)** show the intact protein ESI-MS for P*D and P*DA product formation for the $POP_{169/512Bpy}$-161C/520C(P*) variant. The theoretical masses of PD, PDA, P*D, and P*DA are 72462.14, 72874.14, 72465.12, and 72877.12 Da, respectively. The experimental masses of PD, PDA, P*D, and P*DA from intact protein ESI-MS are 72459, 72864, 72464, and 72878 Da, respectively.

Table S1. Various site pairs distances predicted from the previous MD simulation data.

Site pair	Open distance (Å)	Close distance (Å)	Change in the distance (Δ)
**256/516**	29.2	16.4	12.8
**238/516**	23.5	11.3	12.2
**256/513**	35.3	23.1	12.1
**214/516**	15.8	4.1	11.7
**161/517**	14.3	25.8	11.6
**167/520**	13.7	25.2	11.5
**190/520**	11.8	23.1	11.3
**238/513**	29.3	18.2	11.1
**215/513**	24.1	13.1	11.1

**191/517**	8.6	19.6	11.0
**166/520**	15.8	26.7	11.0

Table S2. Measured FRET efficiency (E_{FRET}) values for different POP variants in the presence or absence of Ni(II) at RT.

Variant	E_{FRET} (-Ni(II))	E_{FRET} (+Ni(II))
$POP_{WT/256C/513C}$	0.42	n/a
$POP_{Bpy167/517/256C/513C}$	0.40	0.44
$POP_{Bpy167/517/161C/520C}$	0.53	0.47
$POP_{Bpy169/512/161C/520C}$	0.50	0.53

$E_{FRET} = 1-(I_{DA}/I_D)$; I_{DA} is the emission intensity of a protein sample labeled with both donor and acceptor dye. I_D is the emission intensity of a protein sample labeled with only the donor dye. [39]

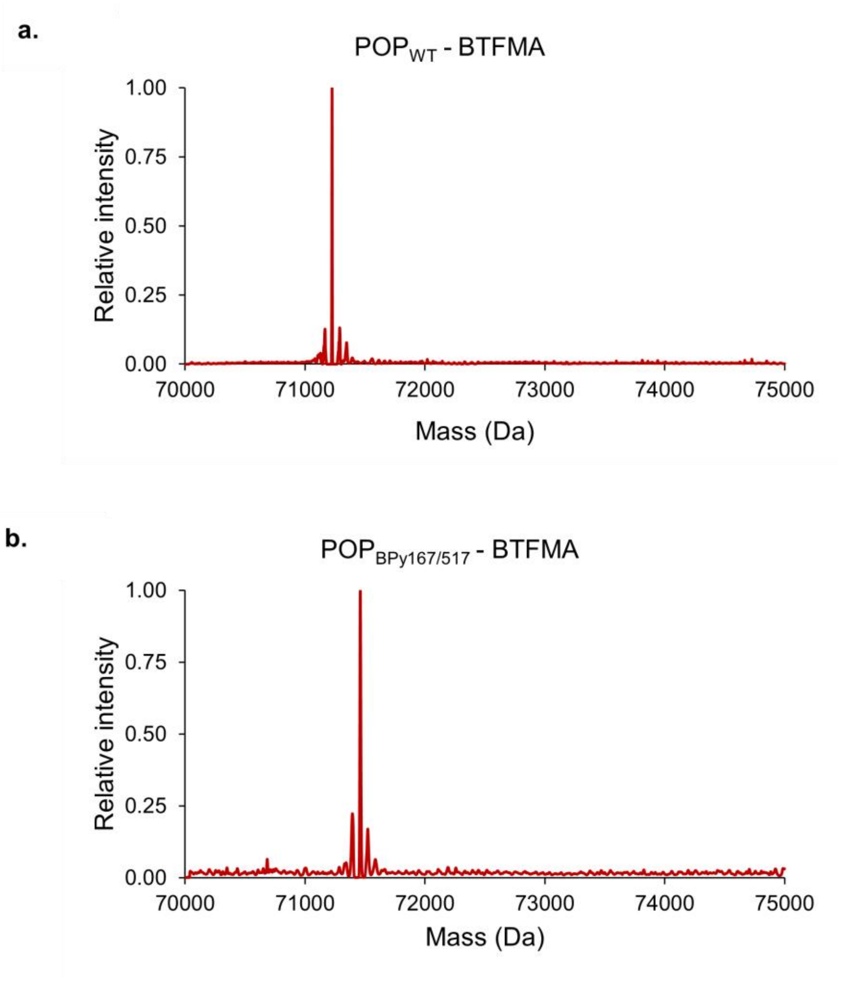

Figure S13. Bioconjugation of ^{19}F NMR probe BTFMA via cysteine acetamide crosslinking at residue 593 **a)** and **b)** intact protein ESI-MS of BTFMA conjugated POP_{WT} and $POP_{Bpy167/517}$, respectively. The theoretical masses of the BTFMA-conjugated proteins (without His-tag) are 71221.12 and 71459.15 Da, respectively. The experimental masses from intact protein ESI-MS are 71225 and 71461 Da, respectively. The bioconjugation efficiency is >99%.

II. Material and methods

A) Materials

All reagents were purchased from commercial suppliers and used as received unless otherwise noted. Primers were purchased from Sigma-Aldrich. DpnI, NcoI, XhoI restriction enzymes, and T4 Ligase were purchased from New England Biolabs, Inc (Ipswitch, MA). PrimeStar Max 2X premix was purchased from Takara Bio Inc (Kusatsu, Shiga Prefecture, Japan). QIAprep Spin Miniprep Kit and QIAquick Gel Extraction Kit were purchased from QIAGEN Inc. (Valencia, CA). DNA Cleanup Kit was purchased from Zymo Research (Irvine, CA). Nickel (II) nitrilotriacetic acid (Ni-NTA) resin, agar, and agarose were purchased from Thermo Fisher. Centrifugal Filters of 30 kDa MW cutoff were purchased from EMD Millipore (Billerica, MA). Pageruler plus pre-stained protein ladder was purchased from Thermo Fisher. 2X Laemmli sample buffer was purchased from Bio-Rad. Dye pair for FRET experiments : Tide Fluor™ 1 maleimide

(EDANS derivative) and Tide Quencher™ 1 maleimide (DABCYL derivative) were purchased from AAT Bioquest (Pleasanton, CA). AZDye 647 maleimide (for the MST experiment) was purchased from Click Chemistry Tools (Scottsdale, Arizona). Mag-fura-2 was purchased from Invitrogen (Waltham, MA). 2-bromo-N-(4-(trifluoromethyl)phenyl)acetamide (BTFMA) was purchased from Oakwood Chemical (South Carolina), Substrate (Z-Ala-Pro-pNA) for POP enzymatic activity was purchased from BACHEM (Bubendorf, Switzerland). Luria broth (LB) and rich medium (2XYT) were purchased from Research Products International (Mt. Prospect, IL). Bio-Rad MicroPulser was used for electroporation. HiTrap Q FF anion exchange column and HiLoad 16/600 superdex 200 pg size exclusion chromatography column were purchased from Cytiva (Marlborough, MA).

B) Methods

Intact protein ESI-MS was performed using Waters Synapt G2S HDMS. A 10-minute LC method (A: H_2O with 0.1 % formic acid, B : acetonitrile with 0.1 % formic acid) with a gradient from 95% A to 1% A over 6 minutes followed by a 4-minutes flush at 95 % A was used with mass spectrometer. The unnatural amino acids R,S-(2,2'-Bipyridin-5-yl)alanine was synthesized according to previously reported protocol by our group.[12] All gene sequencing was done by Quintara Biosciences and whole plasmid sequencing was done by Plasmidsaurus. DNA sequencing results were analyzed in the Benchling. Kinetics data were obtained using a Cary 100 Bio UV-Vis spectrometer by Agilent. MST measurements were done on Monolith NT.115 (Nano Temper). All the absorbance and fluorescence measurements were done on Synergy Neo 2 microplate reader by Agilent except fluorescence measurements in the case of the Mag-fura-2 competition experiment were done on PC1 photon counting spectrophotometer. Protein concentration was determined by Bradford assay/absorbance at 280 nm and DNA concentration was determined by absorbance at 260 nm and the purity was confirmed by ratio of absorbance at 260nm/280nm. SDS-PAGE was used to check the purity of protein samples. 12% SDS-PAGE gels were self-casted according to Biorad protocol. Agarose gels were used to visualize (using ethidium bromide) and purify DNA products. BL21-Gold(DE3), B834(DE3) electrocompetent cells were self-prepared.

C) Software

DynaFit[34,35] was used for fitting the experimental data obtained in the competitive metal chelator binding experiments using the custom DynaFit scripts given below for each experiment :

Script #1:

Script for MF2 binding to Ni (no protein)

MF2 = M

Ni = N

;____

[task]

task = fit

```
   data = equilibria
[mechanism]
 M + N <==> MN : K1 dissoc
[constants]
 K1 = 1 ? ; unit : uM
[concentrations]
 M = 4.9 ; unit : uM
[responses]
 M = 0.2040 ?
 MN = 0.0005 ?
[data]
   variable N
   file ./NiMf2/Input/09292023_MF2_Ni.txt
[output]
   directory ./NiMf2/Output/09292023_MF2_Ni
[end]
```

Script #2:

Global fitting script for MF2 and protein binding competition for Ni

Protein = P ($POP_{Bpy167/517}$)

MF2 = M

Ni = N

```
;____________________________________________________________________
[task]
   task = fit
   data = equilibria
[mechanism]
 P + N <==> PN : K1 dissoc
 M + N <==> MN : K2 dissoc
```

PN + N <==> PNN : K3 dissoc ;(determined from 19F NMR experiment)

[constants]

K1 = 0.0606? ; unit : uM

K2 = 0.219 ; unit : uM (in buffer 25 mM HEPES + 150 mM NaCl, pH = 7.4)

K3 = 66 ; unit : uM

[data]

variable N

file ./NiMf2/Input/09262023_popbpa_MF2_Ni.txt | response M = 0.2040? , MN = 0.0105? | conc. P = 4.8, M = 4.9

file ./NiMf2/Input/100423_popbpa_MF2_Ni.txt | response M = 0.2 ? , MN = 0.0121 ? | conc. P = 7.72, M = 5

file ./NiMf2/Input/100323_popbpa_MF2_Ni.txt | response M = 0.090 ? , MN = 0.0046 ? | conc. P = 4.13, M = 11.09

[output]

directory ./NiMf2/Output/10042023_popbpa_MF2_Ni_global_three

[end]

Script #3 :

Script for P binding to Ni using 19F NMR experiment

Protein = P (POPWT)

Ni = N

;____

[task]

task = fit

data = equilibria

[mechanism]

P + N <==> PN : K1 dissoc

[constants]

K1 = 70 ? ; unit : uM

[concentrations]

P = 157 ; unit : uM

[responses]

P = 0

PN = 0.0063

[data]

variable N

file ./NiMf2/Input/WT_NMR_593C_fitting.txt

[output]

directory ./NiMf2/Output/WT_NMR_593C_fitting.txt

[end]

Script #4 :

Global fitting script for MF2 and protein binding competition to Ni

Protein = P ($POP_{Bpy169/512}$)

MF2 = M

Ni = N

;____

[task]

task = fit

data = equilibria

[mechanism]

P + N <==> PN : K1 dissoc

M + N <==> MN : K2 dissoc

PN + N <==> PNN : K3 dissoc ;(determined from 19F NMR experiment)

[constants]

K1 = 1? ; unit : uM

K2 = 0.219 ; unit : uM (in buffer 25 mM HEPES + 150 mM NaCl, pH = 7.4)

K3 = 66   ; unit : uM

[data]

variable N

file ./NiMf2/Input/062024_POP169_512bpyMF2_Ni.txt | response M = 0.2012 ? , MN = 0.0286 ? | conc. P = 6.60, M = 4.97

file ./NiMf2/Input/070424_POP169_512bpyMF2_Ni.txt | response M = 0.1808 ? , MN = 0.0312 ? | conc. P = 7.50, M = 5.53

file ./NiMf2/Input/062924_POP169_512bpyMF2_Ni.txt | response M = 0.2188 ? , MN = 0.0335 ? | conc. P = 8.04, M = 4.57

[output]

directory ./NiMf2/Output/070924_pop169_512bpy_MF2_Ni_global_three

[end]

Script #5 :

Global fitting script for MF2 and protein binding competition to Ni

Protein = P (POP_{Bpy169})

MF2 = M

Ni = N

;____

[task]

task = fit

data = equilibria

[mechanism]

P + N <==> PN : K1 dissoc

M + N <==> MN : K2 dissoc

PN + N <==> PNN : K3 dissoc ;(determined from 19F NMR experiment)

[constants]

K1 = 1? ; unit : uM

K2 = 0.219 ; unit : uM (in buffer 25 mM HEPES + 150 mM NaCl, pH = 7.4)

K3 = 66 ; unit : uM

[data]

variable N

file ./NiMf2/Input/060524_POP169bpyMF2_Ni.txt | response M = 0.2061 ? , MN = 0.0107 ? | conc. P = 9.53, M = 4.85

file ./NiMf2/Input/061424_POP169bpyMF2_Ni.txt | response M = 0.0813 ? , MN = 0.0103 ? | conc. P = 9.86, M = 12.30

file ./NiMf2/Input/061524_POP169bpyMF2_Ni.txt | response M = 0.2016 ? , MN = 0.0207 ? | conc. P = 8.26, M = 4.96

[output]

directory ./NiMf2/Output/061524_pop169bpy_MF2_Ni_global_three

[end]

Script #6 :

Script for MF2 and protein binding competition to Ni

Protein = P (POP_{Bpy512})

MF2 = M

Ni = N

;____

[task]

task = fit

data = equilibria

[mechanism]

P + N <==> PN : K1 dissoc

M + N <==> MN : K2 dissoc

PN + N <==> PNN : K3 dissoc ;(determined from 19F NMR experiment)

[constants]

K1 = 1 ? ; unit : uM

K2 = 0.219 ; unit : uM (in buffer 25 mM HEPES + 150 mM NaCl, pH = 7.4)

K3 = 66 ; unit : uM

[concentrations]

P = 3.94 ; unit : uM

M = 7.64 ; unit : uM

[responses]

M = 0.131 ?

MN = 0.0218 ?

[data]

```
variable N
file ./NiMf2/Input/061824_POP512bpyMF2_Ni.txt
[output]
directory ./NiMf2/Output/061824_POP512bpyMF2_Ni
[end]
```

Script #7 :

Script for MF2 and protein binding competition to Ni

Protein = P (POP_{Bpy167})

MF2 = M

Ni = N

```
;____________________________________________________________________________
[task]
   task = fit
   data = equilibria
[mechanism]
 P + N <==> PN : K1 dissoc
 M + N <==> MN : K2 dissoc
 PN + N <==> PNN : K3 dissoc ;(determined from 19F NMR experiment)
[constants]
 K1 = 1 ? ; unit : uM
 K2 = 0.219  ; unit : uM (in buffer 25 mM HEPES + 150 mM NaCl, pH = 7.4)
K3 = 66 ; unit : uM
[concentrations]
 P = 5.17 ; unit : uM
M = 4.09 ; unit : uM
[responses]
 M = 0.2444 ?
 MN = 0.0147 ?
[data]
```

variable N

file ./NiMf2/Input/101723_POP167_MF2_Ni.txt

[output]

directory ./NiMf2/Output/101723_POP167_MF2_Ni

[end]

Script #8 :

Script for MF2 and protein binding competition to Ni

Protein = P (POP$_{Bpy517}$)

MF2 = M

Ni = N

;____

[task]

task = fit

data = equilibria

[mechanism]

P + N <==> PN : K1 dissoc

M + N <==> MN : K2 dissoc

PN + N <==> PNN : K3 dissoc ;(determined from 19F NMR experiment)

[constants]

K1 = 1 ? ; unit : uM

K2 = 0.219 ; unit : uM (in buffer 25 mM HEPES + 150 mM NaCl, pH = 7.4)

K3 = 66 ; unit : uM

[concentrations]

P = 5.28 ; unit : uM

M = 4.05 ; unit : uM

[responses]

M = 0.247?

MN = 0.0136?

[data]

variable N

file ./NiMf2/Input/110123_POP517_MF2_Ni.txt

[output]

directory ./NiMf2/Output/110123_POP517_MF2_Ni

[end]

III. Enzyme preparation

A) Cloning of POP variants

Quikchange mutagenesis:

The site-directed mutagenesis using Quikchange was done by using 50 ng of template DNA, 400 nM of both forward and reverse primers, sterilized MQ water, and 2X Primestar max premix.

Thermal cycler conditions :

1) 98 °C-120 seconds (initial denaturation)
2) 98 °C - 10 seconds (denaturation)
3) 62 °C - 15 seconds (annealing)
4) 72 °C - 210 seconds (elongation)
5) Repeat cycles #2 to #4 for 20 times
6) 72 °C - 300 seconds (final elongation)
7) 4 °C - Store

Analytical 1 % agarose gel was done to confirm the amplification of plasmid DNA. DpnI digestion of the nicked DNA obtained from PCR, was done to digest any remaining parental DNA according to the standard kit protocol. The DNA was purified using a PCR product cleanup kit by Zymo and then transformed into electrocompetent cells. DNA sequencing was done to confirm the presence of the desired mutation.

Splicing overlap extension (SOE) PCR:

SOE PCR was used in those cases where more than one mutation was simultaneously introduced in the gene. SOE-PCR involves two steps : fragments generation and fragments assembly. The first step was to generate the fragments in the separate PCR reactions using 50 ng of template DNA, 400 nM of each primer, sterile MQ water and 2X Primestar max premix using the following thermocycler conditions :

1) 98 °C - 120 seconds (initial denaturation)
2) 98 °C - 10 seconds (denaturation)
3) 55 °C - 15 seconds (annealing)
4) 72 °C - variable, ~5 sec/Kb (elongation)
5) Repeat cycles #2 to #4 for 25 times
6) 72 °C - 120 seconds (final elongation)
7) 4 °C - Store

DpnI digestion of each amplified fragment was done to digest any remaining parental DNA according to the standard kit protocol. The size of each fragment was confirmed by either 1 % or

2 % agarose gel depending upon the calculated fragment length (if the fragment length is less than 1 Kb then 2 % agarose gel was preferred to have good separation on gel), and the fragments were purified by gel extraction.

The second step was to assemble the amplified fragments obtained from the first step with a total of 120 ng of fragments in equal molar ratios, 400 nM each of the general forward and general reverse primers, sterilized MQ water, and 2X Primestar max premix. The following conditions were used in the thermocycler :

1) 98 °C - 120 seconds (initial denaturation)
2) 98 °C - 10 seconds (denaturation)
3) 55 °C - 15 seconds (annealing)
4) 72 °C - 15 seconds (elongation)
5) Repeat cycles #2 to #4 25 times
6) 72 °C - 120 seconds (final elongation)
7) 4 °C - store

Analytical 1 % agarose gel was used to confirm the assembly of fragments and the assembly product was cleaned with a PCR product cleanup kit by Zymo.

Restriction digestion and ligation reaction:

Restriction digestion of the mutated insert DNA (obtained from SOE PCR) and pET28a vector was performed separately using a common set of restriction enzymes NcoI and XhoI according to the manufacturer's guidelines. The digested insert DNA was purified by using a PCR clean-up kit and digested vector was cleaned by gel extraction procedure. A ligation reaction was set up using a Quick T4 DNA Ligase kit from NEB at a ratio of 1:10 vector : insert using a standard protocol. Ligated DNA was then purified by PCR clean-up kit and transformed into electrocompetent cells and colonies were analyzed by colony PCR prior to sequencing.

Primers used for FRET and NMR constructs generation by SOE PCR.

Primer name	Primer Sequence (5' – 3')
Flanking-F	GGA GAT ATA CCA TGG AAG ACC
Flanking-R	GTG GTG CTC GAG GGA CAG
E256C-F	CCT GAC CAA ATG CGG CAA AGG
E256C-R	CCT TTG CCG CAT TTG GTC AGG
Y513C-F	CGT TTT CAT AAA CTG TGT ATT GGC TC
Y513C-R	GAG CCA ATA CAC AGT TTA TGA AAA CG
K161C-F	CGC AAA GAA TGC ACG CCG GA TG
K161C-R	CAT CCG GCG TGC ATT CTT TG CG
P520C-F	CTT AGT GGA TTT GCG AAT AC GG
P520C-R	CCG TAT TCG CAA ATC CAC TA AG
M593C-F	CAGGTCACTGCGGCGCGTCGC
M593C-R	GCGACGCGCCGCAGTGACCTG

Quikchange primers used for "His tag removal" variants and cysteine mutation for MST experiment.

Primer name	Primer Sequence (5' – 3')
K548C-F	CCG AAG AAA TGC TAT CCG CCG ACC CTG ATT TAC
K548C-R	GCG GAT AGC ATT TCT TCG GAT CAA CGT TAT GG
His tag remove-F	GTC CCT CGA GTG ACA CCA CCA CCA CCA CC
His tag remove-R	GGT GGT GTC ACT CGA GGG ACA GCG TTT TCA GG

B) Protein expression and lysis

5ml LB media containing appropriate antibiotics (0.05 mg/mL kanamycin ; 0.02 mg/mL chloramphenicol) was inoculated with cells from a glycerol stock of *E. coli* BL21Gold(DE3) containing the appropriate plasmids (pET28a containing POP gene and bpyala-pEVOL for UAA incorporation). After O/N growth at 37 °C, this culture was then used to inoculate 500 mL of 2XYT media (with required antibiotics), and the cells were cultured at 37 °C, with shaking at 250 rpm until the OD_{600} reached between 0.7 and 0.8. To induce overexpression of the tyrosyl tRNA synthetase gene (present in pEVOL), 20 % (w/v) L-arabinose was added to the culture. This arabinose solution also contained R, S-(2,2'-Bipyridin-5-yl) alanine (1 mM final concentration). To induce overexpression of the POP gene, IPTG (1mM final concentration) was added, and the cells were cultured overnight at 37 °C, 250 rpm. The cells were transferred to centrifugation buckets and pelleted via centrifugation at 3,600 rpm for 30 minutes. The supernatant was discarded, and the pellets were resuspended in 50 mL equilibration buffer. The composition of the equilibration buffer depends upon the type of purification that needed to be done for example, for Ni-NTA purification equilibration buffer consisted of 20 mM Na_2HPO_4, 300 mM NaCl, 10 mM imidazole, pH 7.4 whereas for ion exchange purification equilibration buffer was 20 mM Na_2HPO_4, pH 6.5. The cells were kept at -80 °C until lysis. Cells were sonicated to break the cell wall, the lysed cells were heated at 75 °C for 15 minutes, and the cell debris was pelleted via centrifugation at 12,000 rpm, 4 °C for 30 minutes to obtain the clear lysate.

C) Protein purification

Ni-NTA affinity chromatography:

The supernatant (lysate) was loaded onto Ni-NTA resin. The resin was washed with wash buffer (20 mM Na2HPO4, 300 mM NaCl, 20 mM imidazole, pH 7.4). The protein was eluted using elution buffer (20 mM Na2HPO4, 300 mM NaCl, 250 mM imidazole, pH 7.4). The purity of the protein samples was analyzed by 12 % SDS PAGE. Fractions containing protein were combined and concentrated to a volume less than 1 mL using 30 kDa MW cutoff centrifugal filters and were buffer exchanged in the required buffer according to the application.

Ion-exchange chromatography (IEX):

In the case of variants without His tag, IEX chromatography (using an anion exchanger) was performed as the first protein purification step in place of Ni-NTA purification. Three Hi trap QFF columns (each column volume 5ml) were used in tandem on the AKTA system. The lysate was loaded onto the column by using the super loop (maximum capacity 50 ml) at the flow rate of 4

ml/min. After this, washing was done using 5 CV of buffer A (20 mM Na_2HPO_4, pH 6.5) and then elution was done over 20 CV using optimized 13 % buffer B (20 mM Na_2HPO_4, 1M NaCl, pH 6.5) isocratic condition. Several small elution fractions were collected using an automatic fraction collector and were analyzed using 12 % SDS PAGE. Fractions containing pure protein were combined and concentrated to a volume less than 1 mL using 30 kDa MW cutoff centrifugal filters and buffer exchanged in the required buffer according to the application.

In the case of BpyAla POP variants, the concentrated protein sample was heated with 5 mM of 1,10 phenanthroline at 65 °C for an hour to remove the bound iron metal ion from the protein sample and then the SEC was done to separate the desired iron-free protein from the mixture of excess phenanthroline, iron-phenanthroline complex and aggregates.

Size exclusion chromatography (SEC):

A size-exclusion column (SEC) was used on the FPLC system. A HiLoad 16/600 Superdex 200 pg column was equilibrated with one column volume (CV) of sterile-filtered MilliQ H_2O followed by 2 CVs of sterile-filtered 150 mM NaCl solution (in the case of bpyala variants 50 mM EDTA + 150 mM NaCl, pH 8.0 buffer was used). The flow rate was set to 1 mL/min and fractions were collected. To analyze the quality of the protein samples, SDS-PAGE and dynamic light scattering were used.

IV. Biophysical techniques

MST :
The labeled protein sample was incubated with 16 different dilutions of ligand in small PCR tubes for 15 minutes at 55℃. The final concentration of the labeled protein was 100 nM and different range of ligand concentrations (pM – mM) were tried in each trial. After this capillary scan was done followed by which MST measurements were done with 20 % LED power and different MST powers 20 %, 40 %, 80 %.

ITC :
The standard protocol for the nano ITC (from TA instruments) was followed. The concentration of the protein sample was ~ 50 µM and different equivalents of metal (like 10 eq, 20 eq, 50 eq) was titrated in different trials. The measurements were done at 55℃, 350 rpm with total of 30 injections of $NiCl_2$ (each injection 1.5 µl). The time spacing between each injection was 120 sec.

Mag-fura-2 (MF2) competition experiment :
The experiments were conducted in a 3 ml cuvette on PC1 photon counting spectrofluorometer with stirring on. The excitation spectrum was scanned from 270 nm to 450 nm at an emission wavelength of 505 nm. The chelex treated 25 mM HEPES+150 mM NaCl, pH 7.4 buffer was used. The concentration of MF2 was quantified using the reported extinction coefficient of 22,000 M^{-1} cm^{-1} at 366 nm.[32] The concentration of protein was quantified from both Bradford assay and A280 nm, the concentration values were very close from both the methods. In three trials, different equivalents of protein : MF2 were titrated (1:1, 2:1,1:2) with increasing amount of Ni(II) from 0 to 20 µM. In each addition 1 µM of Ni(II) was added and mixed well using the pipette. The sample was allowed to equilibrate at RT (with stirring on) for 1.5 minutes between each addition before taking the fluorescence measurement. For plotting the data of fluorescence intensity versus Ni(II),

the intensity values at 372 nm excitation wavelength were taken as that was the λmax in the excitation spectra. K_d determination was done by fitting the data using DynaFit software.

Bioconjugation of FRET dye pair :

After SEC purification, the protein was buffer exchanged into 50 mM HEPES pH 7.4. Prior to labeling, the protein was treated with a 10-fold excess of TCEP for 10 minutes at 750 rpm, RT. 5 mM stock solution of dyes was prepared in DMSO. The final concentration of protein in the reaction mixture was 25 µM. The molar ratio for the single dye bioconjugation was protein:dye:1:2 (donor or acceptor) whereas for double bioconjugation the ratio was protein: donor dye: acceptor dye:1:2:10. The final concentration of cosolvent DMSO was 10 %. In the case of double bioconjugation dyes were added in sequential manner so firstly donor dye was added and after the completion of first reaction acceptor dye was added. The reaction was done at 750 rpm, RT for 30 minute. The progress of the reaction was monitored by protein LCMS. After the reaction was completed, the unreacted dye was quenched by a 10-fold excess of glutathione (to the total moles of dye added) and finally, buffer exchanged (using 30 kDa MW cutoff spin filters) into 50 mM HEPES, pH 7.4 buffer.

Bioconjugation of BTFMA for ^{19}F NMR:

Following SEC purification, the protein was buffer-exchanged into 25 mM HEPES, 150 mM NaCl, pH 7.4. BTFMA was prepared as a 10 mM stock solution in DMSO and added to the labeling reaction at a 3-fold molar excess relative to the protein. The final protein concentration in the reaction was 50 µM. Labeling was carried out at room temperature in a 1 mL reaction volume with agitation at 750 rpm. Reaction progress was monitored by protein LC-MS, and excess BTFMA was removed via buffer exchange post-reaction.

Bioconjugation of dye for MST :

After SEC purification, the protein was buffer exchanged into the labeling buffer (17.7 mM NaH2PO4, 32.5 mM Na2HPO4, 100 mM NaCl, pH 7). 500 µM stock solution of AZDye 647 maleimide was prepared in DMSO. The reaction was performed at RT under dark condition for 30 minutes. The final ratio of protein : dye was 1 : 3 (5 % DMSO) and the final concentration of protein was 10 µM. Bioconjugation efficiency was confirmed by protein LCMS. Excess free dye was quenched by a 10-fold excess of glutathione and finally buffer exchanged (using 30 kDa MW cutoff spin filter) into 50 mM HEPES, pH 7.4.

Kinetic assay :
The hydrolysis of benzyloxycarbonyl-alanyl-prolyl-p-nitroanilide (Z-Ala-Pro-pNA) by POP enzyme was monitored at 410 nm, 85 °C.[12] Because the pNA (para nitro aniline) is chromogenic (yellow) in nature, the initial rate was determined spectrophotometrically by measuring the amount of pNA produced. According to the previously reported protocol by our group[12], 900µl master solution (containing HEPES buffer pH 7.4, NaCl, enzyme, and EDTA/$NiCl_2$) was first added to a quartz cuvette, and the cuvette was incubated at 85 °C in the spectrophotometer for 1 minute. To start the reaction, substrate and DMSO were added. The final volume of the reaction mixture was 1 ml, and the final concentration of each component was 30 mM HEPES (pH 7.4), 800 mM NaCl,

10 nM enzyme, 5 µM $NiCl_2$/1 mM EDTA, 0.01-1.00 mM substrate, and 10% (v/v) DMSO. The absorbance was monitored every 6 seconds for 1 minute and initial rates were determined by converting absorbance values to concentration using the molar extinction coefficient of pNA (7,126 $M^{-1}cm^{-1}$). Origin Pro was used to plot the curve between the initial rate and substrate concentration. The curve was fitted nonlinearly using the Michaelis-Menten equation in Origin Pro 2021.

^{19}F NMR:

NMR samples contained 0.084-0.157 mM BTMFA-labeled protein, 25 mM HEPES, 150 mM NaCl, and 10 % v/v D2O at pH 7.4, with 0.22 mM 2,2-dimethyl-2-silapentanesulfonic acid (DSS) and 0.022 mM trifluoroacetate as internal references. Nickel chloride was added as 0.25, 0.5, 0.75, 1, 2, 3, and 10 molar equivalents. NMR spectra were collected on a Bruker Avance Neo II 14.1 T (600 MHz proton frequency) spectrometer equipped with an HCN cryogenic probe tuned to the fluorine frequency. All spectra were collected at 25 ºC. Free induction decays were acquired with 2048 scans, recovery delay of 1 second, acquisition time of 0.15 s, and a 90º pulse length of 16 µs. Spectra were processed using MestReNova, with zero-filling to 8192 points and apodization of 20 Hz exponential line broadening.

V. Synthetic methods and materials

Unless otherwise noted, all reagents were obtained from commercial suppliers and used without further purification. Deuterated solvents were obtained from Cambridge Isotope Laboratories, Inc (Tewksbury, MA). Silicycle silica gel plates (250 mm, 60 F254) were used for analytical TLC, and preparative chromatography was performed using SiliCycle (Quebec City, QC) SiliaFlash silica gel (230-400 mesh). Column chromatography was carried out using Silicycle 230-400 mesh silica gel. ^{1}H was recorded at 400 MHz on a Varian 400 MHz Inova NMR Spectrometer and chemical shifts are reported relative to residual solvent peaks. Chemical shifts are reported in ppm and coupling constants are reported in Hz.

Synthesis of Bipyridyl alanine (BpyAla) unnatural amino acid:

R,S-(2,2'-bipyridin-5-yl)alanine·3HCl was synthesized using a protocol modified from reported methods.[12,44]

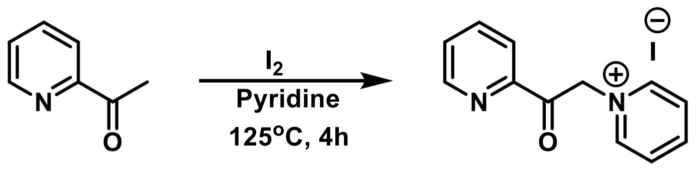

1-(2-pyridylacetyl)pyridinium iodide :

In a clean dry 1L Round bottom flask was added Iodine (75.6 g, 300 mmol) which was dissolved in pyridine 400 mL. Acetyl pyridine (33.6 mL, 300mmol) was added to the solution and solution was heated to 125ºC for 4 hours. Heating was turned off and solution was allowed to stir as it cooled overnight. The suspension was filtered under vacuum ,washed with EtOH (100 mL, then

50 mL) and dried over vacuum giving the crude product as a black solid (98 g, qt. yield). The product was used for the next step of the synthesis without any further purification.

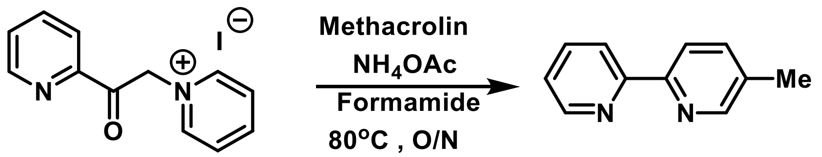

5-methyl-2,2'-bipyridine:

A solution of 1-(2-pyridylacetyl)pyridinium iodide (49 g, 150 mmol) in formamide (300 mL) was treated sequentially with methacrolein (12.4 mL, 150 mmol) and ammonium acetate (34.65 g, 450 mmol). The reaction mixture was stirred at 80 °C overnight, then cooled to room temperature and extracted with dichloromethane. The combined organic extracts were washed with brine (500 mL), dried over anhydrous $MgSO_4$, filtered, and concentrated under reduced pressure. The crude material was purified by silica gel column chromatography (5% MeOH in CH_2Cl_2) to afford the desired product as a yellow oil (10.3 g, 40%). The spectral data were consistent with those reported in the literature.[44]

^{1}H NMR (400 MHz, cdcl$_3$) δ 8.63 – 8.59 (m, 1H), 8.45 (d, *J* = 2.3 Hz, 1H), 8.31 (dt, *J* = 7.9, 1.1 Hz, 1H), 8.24 (d, *J* = 8.0 Hz, 1H), 7.73 (td, *J* = 7.7, 1.9 Hz, 1H), 7.55 (dd, *J* = 8.2, 2.4 Hz, 1H), 7.24 – 7.17 (m, 1H), 2.31 (s, 3H).

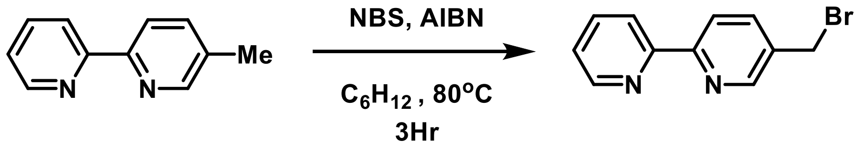

5-bromomethyl-2,2-bipyridine:

5-Methyl-2,2′-bipyridine (10.3 g, 60.6 mmol) was dissolved in cyclohexane (300 mL), and N-bromosuccinimide (NBS, 20.5 g, 116 mmol) was added. The solution was heated to 80 °C, and azobisisobutyronitrile (AIBN, 542 mg, 3.3 mmol) was introduced portionwise over 20 min. The mixture was maintained at 80 °C for an additional 3 h, during which a black precipitate formed. The reaction mixture was cooled slightly, and the supernatant was decanted from the precipitate. The solid was rinsed with hot cyclohexane (2 × 30 mL), and the combined filtrates were concentrated under reduced pressure to afford a yellow–brown oil. The residue was placed in an ice bath with stirring, and cold hexane (50 mL) was added in one portion, resulting in the formation of a pale-yellow precipitate. The solid was collected by vacuum filtration, washed with cold hexane (2 × 20 mL), and dried under vacuum to yield the product as a pale-yellow solid (7.3 g, 49%).

^{1}H NMR (400 MHz, cdcl$_3$) δ 8.68 (d, *J* = 3.1 Hz, 1H), 8.40 (d, *J* = 8.1 Hz, 1H), 7.83 (ddd, *J* = 16.5, 8.2, 2.1 Hz, 1H), 7.36 – 7.26 (m, 1H), 4.54 (s, 2H).

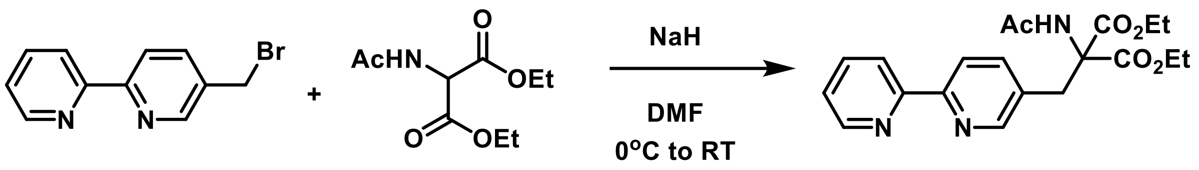

Diethyl 2-(2,2′-bipyridin-5-ylmethyl)-2-acetamidomalonate:

To a 250 mL round-bottom flask containing NaH (1.3 g, 55 mmol, 60% dispersion in mineral oil) at 0 °C was added anhydrous DMF (6 mL). A solution of diethylacetamidomalonate (6.38 g, 29.6 mmol) in anhydrous DMF (12 mL) was added dropwise with stirring. After addition, the cooling bath was removed and the mixture was stirred at room temperature for 30 min. The reaction was then cooled again to 0 °C, and a solution of bromomethyl-2,2′-bipyridine (7.3 g, 29.6 mmol) in DMF (6 mL) was added dropwise. The mixture was stirred at room temperature for 5 h, cooled to 0 °C, and quenched by the addition of water (100 mL) with vigorous stirring. The resulting precipitate was collected by vacuum filtration, dissolved in $CHCl_3$ (50 mL), and treated with activated charcoal (ca. 2.5 g). The suspension was filtered through Celite, and the filtrate was concentrated under reduced pressure to afford the product as a white solid (7.0 g, 61%). Analytical data were consistent with literature values.[12]

^{1}H NMR (400 MHz, $cdcl_3$) δ 8.67 (ddd, J = 4.8, 1.8, 0.9 Hz, 1H), 8.37 – 8.31 (m, 2H), 8.29 (d, J = 8.1 Hz, 1H), 7.81 (td, J = 7.7, 1.8 Hz, 1H), 7.47 (dd, J = 8.1, 2.3 Hz, 1H), 7.30 (ddd, J = 7.5, 4.8, 1.2 Hz, 1H), 6.59 (s, 1H), 4.29 (qd, J = 7.1, 2.4 Hz, 4H), 3.74 (s, 2H), 2.07 (s, 3H), 1.31 (t, J = 7.1 Hz, 6H).

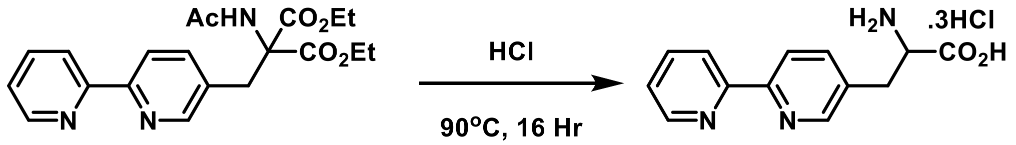

(2,2’-bipyridin-5-yl)alanine·3HCl:

A suspension of diethyl-2-(2,2′-bipyridin-5-ylmethyl)-2-acetamidomalonate (7.0 g, 18 mmol) in concentrated HCl (60 mL, 12 N) was heated at reflux overnight. The reaction mixture was concentrated under reduced pressure to afford R,S-(2,2′-bipyridin-5-yl)alanine as a pale-yellow hydrochloride salt.

^{1}H NMR (400 MHz, d_2o) δ 8.64 (d, J = 5.7 Hz, 1H), 8.58 (s, 1H), 8.41 (dt, J = 17.1, 8.2 Hz, 2H), 8.14 (d, J = 8.3 Hz, 1H), 7.98 (s, 1H), 7.83 (t, J = 6.8 Hz, 1H), 4.25 (s, 1H), 3.36 – 3.20 (m, 2H).

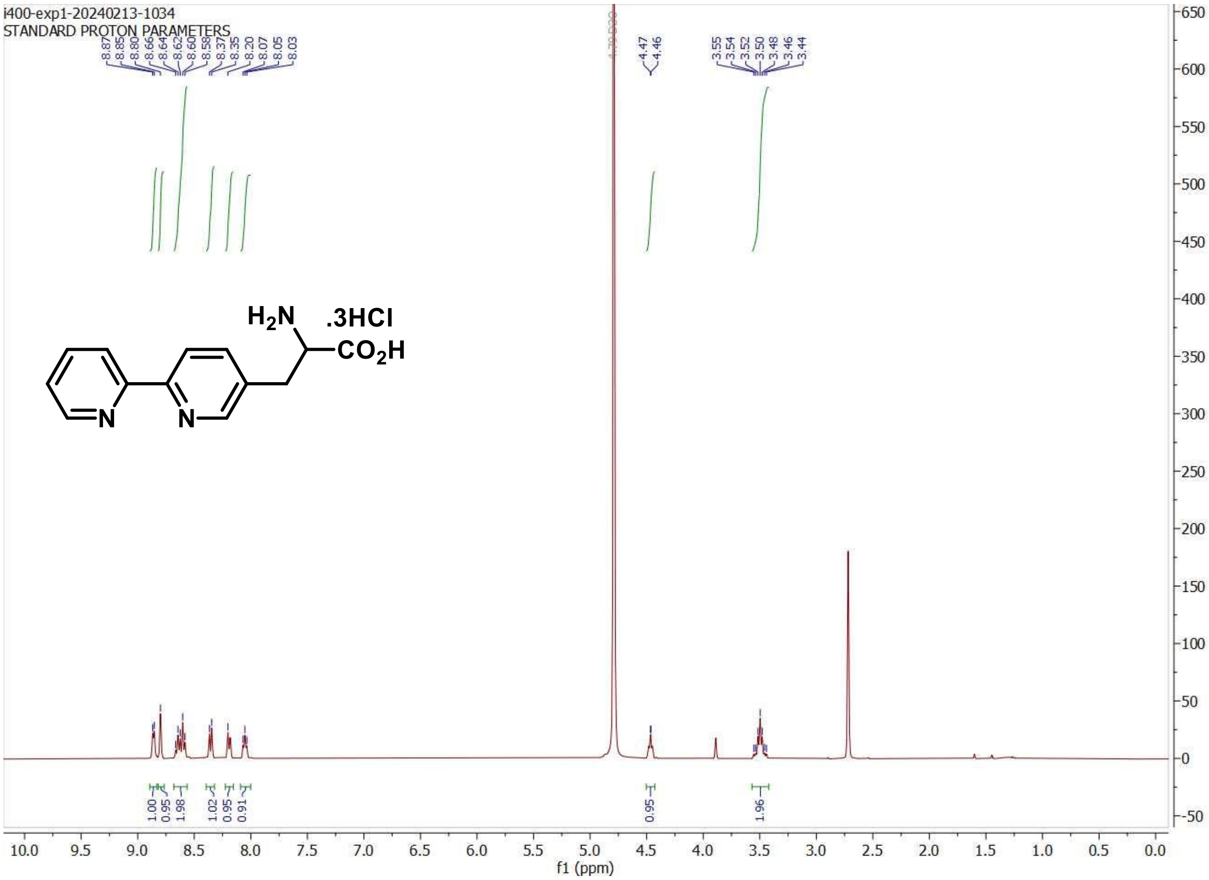

VI. Computational methods

Simulation Method:

The simulations were performed with the CHARMM36 force field for water, ions, and natural amino acids, with custom parameters for the bipyridine-added amino acids was initially modeled from *CGENFF* and then further modified. The non-bonded interactions of the metal (Cu^{2+}, Zn^{2+}, Ni^{2+}) with the Bpy groups were adjusted via the Lennard-Jones potential NBFIX option of CHARMM to match the experimental binding constants to one and two BPY (supplementary information). The system consists of 10037 protein atoms in a 95 Å × 108 Å × 104 Å orthorhombic water box consisting of 32690 water molecules, 92 Cl^- ions, 100 K^+ ions, and when appropriate a single Ni^{2+} ion. The simulation systems were generated, and initially relaxed in an NPV ensemble, and then equilibrated in an NPT ensemble with temperature set to 300 K and the isotropic pressure set to 1 atm. Simulations were run in the NPT ensemble with periodic boundary conditions. Temperature was controlled to 300 K with a Langevin thermostat, and pressure was controlled to 1 atm using Langevin piston barostat. The integration was performed with a time step was 2 fs, and bonds involving hydrogen atoms were held rigid with constraints enforced by the RATTLE algorithm. Long-distance van der Waals interactions were switched off smoothly between 10 and 12 Å, and electrostatic interactions were calculated using the particle-mesh Ewald method. Two separate replica-exchange umbrella sampling calculations were performed. The PMF in the presence of the Ni(II) was calculated using umbrella sampling from 180 windows simulations (150

ns/window) biased by a 125 kcal/mol/Å^2 harmonic potential with reference distances ranging from 2 Å to 37.8 Å with a separation of 0.2 Å. Ni(II) was restrained to Bpy167 by a half-harmonic potential at 5 Å from the center of the two Bpy nitrogen atoms. The PMF calculation in the absence of Ni^{2+} had 196 windows (28 ns/window) with centers ranging from 0 Å to 39 Å. The N-N distance is measured between the centers of mass of the two nitrogen atoms on Bpy167 and the two nitrogen atoms on Bpy517. Postprocessing of the biased data to reconstruct the unbiased free energy landscape was done using the Weighted Histogram Analysis Method (WHAM).[43] For the simulations on the S477-H592 distance, each window was run as a single trajectory, with the methods otherwise the same as the REUS simulations, and the PMFs were calculated as the logarithms of the histograms.

Force field parameters for bipyridine (Bpy) and divalent cations :

The equilibrium binding constant of one divalent ion with one or two Bpy are reported in reference.[45] The models were developed for the CHARMM36 force field[46–49] for water, ions, and natural amino acids, with custom parameters for the bipyridine-added amino acids was initially generated via CGENFF[50] and were then further modified. The non-bonded interactions of the metal (Cu^{2+}, Zn^{2+}, Ni^{2+}) with the Bpy groups were adjusted via the Lennard-Jones potential NBFIX option of CHARMM[51] to match the experimental binding constants to one and two Bpy. To accurately model the binding ability of Bpy in the present of divalent cations (Ni^{2+}, Zn^{2+}, etc.), we carefully parametrized the interaction between Bpy and those cations in the force field. To do that, we carried out PMF calculations from MD simulations of Bpy molecules and divalent cations in solution to calibrate the pair-wise specific Lennard-Jones (LJ) interactions via the NBFIX parameter option of the CHARMM force field. To minimize the disruption in the force field, only the pairwise parameters of the metal with the nitrogen atom NG2R60 were modified. Taking Ni^{2+} as an illustrative example, the $\log_{10}$ of the equilibrium constant of the binding reaction of one ion with one and two Bpy are reported to be 7.06 and 14.0, respectively, at 25 °C and 1.0 atm.[45] More specifically, the equilibrium reactions are expressed as,

$$Ni^{2+} + Bpy \Leftrightarrow Ni(Bpy)^{2+}$$

where

$$K_{\mathrm{eq}}^{(1)} = \frac{[\mathrm{Ni(Bpy)}^{2+}]}{[\mathrm{Ni}^{2+}][\mathrm{Bpy}]}$$

with $\log_{10}\left(K_{\mathrm{eq}}^{(1)}\right) = 7.06$, and

$$Ni^{2+} + 2\ Bpy \Leftrightarrow Ni(Bpy)^{2+} + Bpy \Leftrightarrow Ni(Bpy)_2{}^{2+}$$

where

$$K_{\mathrm{eq}}^{(2)} = \frac{[\mathrm{Ni(Bpy)_2^{2+}}]}{[\mathrm{Ni^{2+}}][\mathrm{Bpy}]^2} = \frac{[\mathrm{Ni(Bpy)^{2+}}]}{[\mathrm{Ni^{2+}}][\mathrm{Bpy}]} \times \frac{[Ni\mathrm{(Bpy)_2^{2+}}]}{[\mathrm{Ni(Bpy)^{2+}}][\mathrm{Bpy}]} = K_{\mathrm{eq}}^{(1)} \cdot K_{\mathrm{eq}}^{*}$$

where $\log_{10}\left(K_{\mathrm{eq}}^{(2)}\right) = 14.0$. It follows that the association of one metal to one Bpy that is already coordinated to one additional metal in solution,

$$\mathrm{Ni(Bpy)^{2+} + Bpy \Leftrightarrow Ni(Bpy)_2{}^{2+}}$$

has this equilibrium constant

$$K_{\mathrm{eq}}^{*} = \frac{[\mathrm{Ni(Bpy)_2^{2+}}]}{[\mathrm{Ni(Bpy)^{2+}}][\mathrm{Bpy}]} = \frac{K_{\mathrm{eq}}^{(2)}}{K_{\mathrm{eq}}^{(1)}},$$

Here, $\log_{10}\left(K_{\mathrm{eq}}^{*}\right) = \log_{10}\left(K_{\mathrm{eq}}^{(2)}\right) - \log_{10}\left(K_{\mathrm{eq}}^{(1)}\right) = 14.0 - 7.06 = 6.94.$

Following this analysis, we calculated the association constants from the potential of mean force between the two binding partners using the expression,[52]

$$K_{\mathrm{eq}} = \frac{1}{1660} \int_0^{R_c} 4\pi r^2 \, \mathrm{e}^{-W(r)/k_{\mathrm{B}}T} dr \qquad (1)$$

where r is the distance between the 2 binding partners, $W(r)$ is the one-dimensional PMF, such that the radial PMF, $W(r)$, goes to zero at large distance, $k_{\mathrm{B}}T = 0.593$ kcal/mol, and 1660 is the pre-factor of unit conversion from Å^3 to mole per liter. This expression can be used to calculate both $K_{\mathrm{eq}}^{(1)}$ from the separation PMF of one Bpy and one metal in solution, and K_{eq}^{*} from the separation PMF of one Bpy in the presence of one metal pre-coordinated to one additional Bpy in solution (Figure S14).

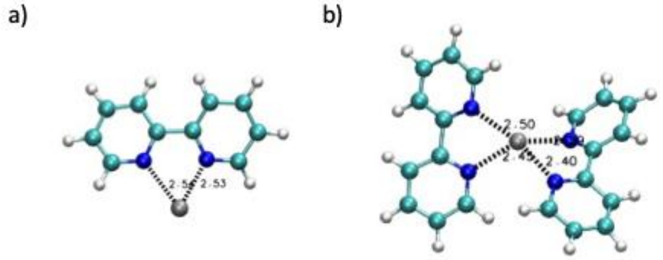

Figure S14. Configurations of Ni^{2+} coordinated by one **(a)** and two **(b)** Bpy molecules in aqueous solution. These initial configurations were used for the calculation of the PMFs shown in Figure S16.

Testing different combinations of NBFIX parameters, we acquired one-dimensional PMFs for those NBFIX parameters (Figure S15), calculated the equilibrium binding constant for each PMF, and identified the optimal NBFIX parameters. The same procedure was used for all 3 divalent

cations. The results with the optimized set of NBFIX parameters yielding results closest to the experimental values for the divalent cations (Ni^{2+}, Zn^{2+}, Cu^{2+}) and Bpy in solution in Table S3. The force field parameter files are provided as supplementary information.

Table S3. Divalent metal binding to Bpy molecules in solution

	Ni^{2+}		Zn^{2+}		Cu^{2+}	
	Exp[a]	Comp[b]	Exp[a]	Comp[b]	Exp[a]	Comp[b]
$\log_{10}\left(K_{\mathrm{eq}}^{(1)}\right)$	7.06	7.725	5.34	5.566	8.52	8.888
$\log_{10}\left(K_{\mathrm{eq}}^{*}\right)$	6.94	8.108	4.62	7.044	5.78	7.422
NBFIX Emin (NG2R60)[c]		-10.0		-10.5		-9.0
NBFIX Rmin (NG2R60)[d]		2.0		2.5		2.05

[a]Experimental values are taken from reference.[45] [b]Computational results were calculated using optimized NBFIX parameters given in the Table as described in the method. [e]The NBFIX parameter Emin is in kcal/mol. [f]The NBFIX parameter Rmin is in Å.

PMF calculations:

We generated 24 water boxes with dimension of 50 x 50 x 50 $Å^3$ which contain 3901 water molecules and 1 Bpy molecule for 1 Ni^{2+} and 2 Cl^- (to keep the electronically neutralization of the system) for deriving $K_{\mathrm{eq}}^{(1)}$. Replicas-exchange umbrella sampling (RE-US) MD were used to calculate the PMF with 24 US windows, each window represents the generated water box which the distance between the center of mass of the two nitrogen atoms of the Bpy molecule and the Ni^{2+} parting further away 0.3 Å consecutively up to a distance of 7.2 Å. We generated 24 water boxes with dimension of 50 $\times$ 50 $\times$ 50 $Å^3$ which contain 3890 water molecules and 2 Bpy molecules for 1 Ni^{2+} and 2 Cl^- with constraint (the restraint force constant is 1.0 kcal/mol/$Å^2$) on the position of one heavy atoms of the Bpy molecule and the Ni^{2+} are in their most stable position derived from $K_{\mathrm{eq}}^{(1)}$, no constraint on the other Bpy molecule for deriving K_{eq}^{*}. Also, RE-US MD on the 24 windows, each window represents the generated water box which the distance between the center of mass of the two nitrogen atoms in the free Bpy molecule and the Ni^{2+} parting further away 0.3 Å consecutively up to 7.2 Å. Finally, we performed a 2 ns production RE-US MD run on both the 24 windows at 300 K by using the harmonic force constant of 125 kcal/mol/$Å^2$ as restraint between Ni^{2+} and the free Bpy molecule. The Langevin thermostats with a damping coefficient of 1 ps^{-1} are used to keep the temperature constant. The cutoff of the van der Waals interactions and short-range electrostatic interactions were set to ~12 Å. Long range electrostatic interactions were handled with the particle meshd (PME) method.[53] The Weighted Histogram Analysis Method (WHAM)[43] was used for postprocessing the information from biased US to reconstruct the unbiased PMFs. In WHAM, the bin size was set to 0.02 Å. The tolerance for

iteration was set to 0.00001. The temperature was set to 300 K. The simulation were carried out with the multiple copy algorithm[54] implemented in the program NAMD.[55] All PMFs were corrected with the Jacobian $2k_{\mathrm{B}}T\ln(r)$ term, so that all PMFs go to a constant at a large distance. All the PMFs are shown in Figure S15.

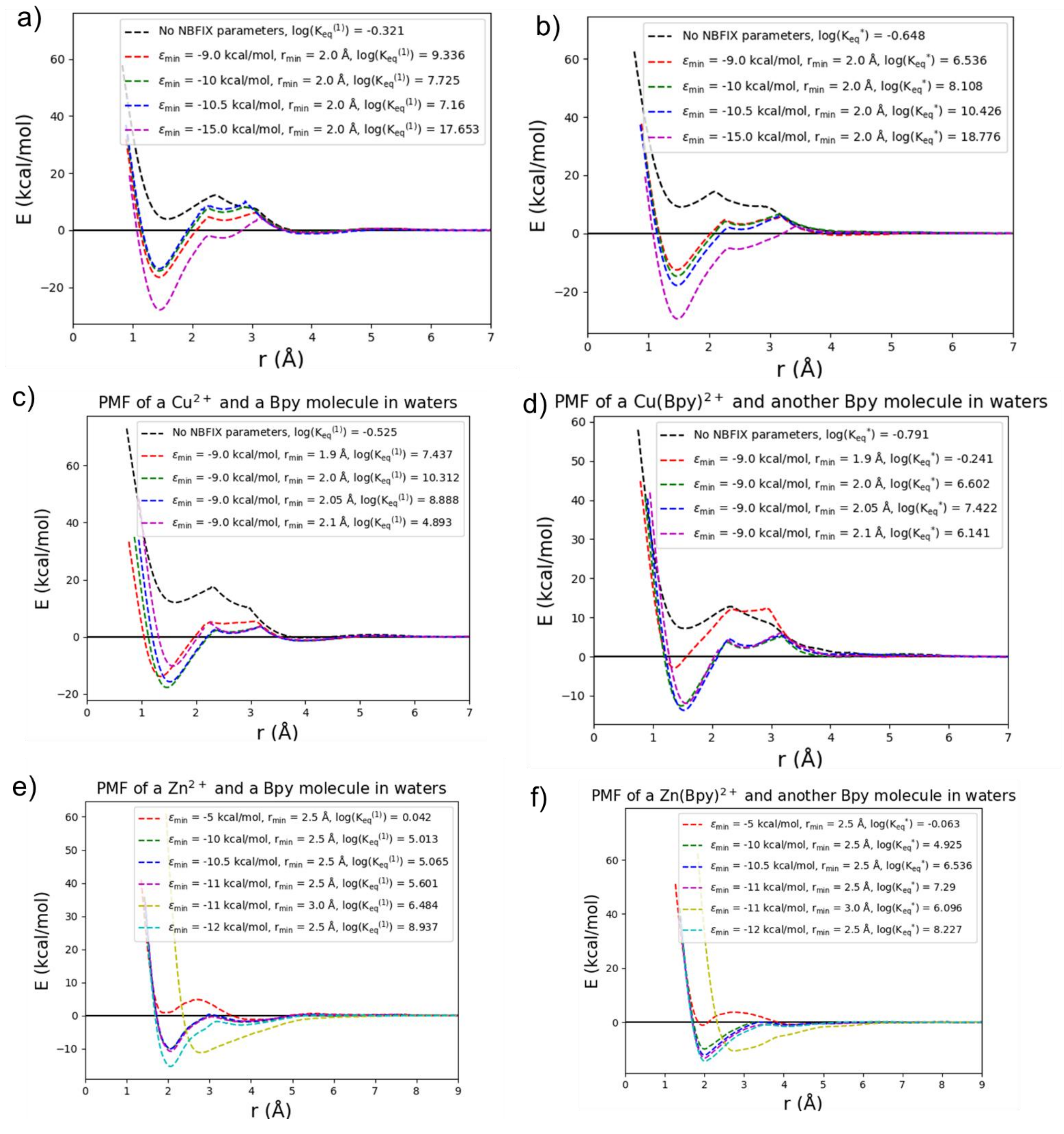

Figure S15. PMFs of the divalent cation with one and two Bpy molecules in aqueous solution for different combinations of NBFIX parameters. **a)** PMFs of one Bpy molecule with one Ni^{2+} cation in solution, **b)** PMF of one Bpy molecule with one Ni^{2+} cation coordinated to one Bpy molecule in solution, **c)** PMF of one Bpy molecule with one Cu^{2+} cation in solution, **d)** PMF of one Bpy molecule with one Cu^{2+} cation coordinated to one Bpy molecule in solution, **e)** PMF of one Bpy molecule with one Zn^{2+} cation in solution, and **f)** PMF of one Bpy molecule with one Zn^{2+} cation coordinated to one Bpy molecule in solution. All PMFs were corrected with Jacobian $2k_BT\ \ln(r)$ term to become constant at a large distance.

Material availability: All the files needed for the MD simulations of the system are available at this link : https://github.com/RouxLab/bpy-simulation-files

VII. References

12 Y. S. Zubi, K. Seki, Y. Li, A. C. Hunt, B. Liu, B. Roux, M. C. Jewett and J. C. Lewis, "Metal-responsive regulation of enzyme catalysis using genetically encoded chemical switches", *Nat Commun.*, 13, 1864 (2022).

32 A. M. Sydor, J. Liu and D. B. Zamble, "Effects of metal on the biochemical properties of Helicobacter pylori HypB, a maturation factor of [NiFe]-hydrogenase and urease", *J Bacteriol.*, 193, 1359–1368 (2011).

34 P. Kuzmič, "DynaFit--a software package for enzymology", *Methods Enzymol.*, 467, 247–280 (2009).

35 P. Kuzmič, "Program DYNAFIT for the analysis of enzyme kinetic data: application to HIV proteinase", *Anal Biochem.*, 237, 260–273 (1996).

39 L. D. Saraswat and S. Lowey, "Subunit Interactions within an Expressed Regulatory Domain of Chicken Skeletal Myosin", *Journal of Biological Chemistry*, 273, 17671–17679 (1998).

43 Grossfield, Alan, "WHAM: the weighted histogram analysis method", version 2.1.

44 E. Klemencic, R. C. Brewster, H. S. Ali, J. M. Richardson and A. G. Jarvis, Using BpyAla to generate copper artificial metalloenzymes: a catalytic and structural study", *Catal. Sci. Technol.*, 14, 1622–1632 (2024).

45 R. M. Smith and A. E. Martell, "Critical Stability Constants, Vol. 2 (Amines)", *Plenum Press, New York*, 2, (1975).

46 A. D. MacKerell, D. Bashford, M. Bellott, R. L. Dunbrack, J. D. Evanseck, M. J. Field, S. Fischer, J. Gao, H. Guo, S. Ha, D. Joseph-McCarthy, L. Kuchnir, K. Kuczera, F. T. K. Lau, C. Mattos, S. Michnick, T. Ngo, D. T. Nguyen, B. Prodhom, W. E. Reiher, B. Roux, M. Schlenkrich, J. C. Smith, R. Stote, J. Straub, M. Watanabe, J. Wiórkiewicz-Kuczera, D. Yin

and M. Karplus, “All-atom empirical potential for molecular modeling and dynamics studies of proteins”, *J Phys Chem B.*, 102, 3586–3616 (1998).

47 R. B. Best, X. Zhu, J. Shim, P. E. M. Lopes, J. Mittal, M. Feig and A. D. MacKerell, “Optimization of the additive CHARMM all-atom protein force field targeting improved sampling of the backbone φ, ψ and side-chain χ(1) and χ(2) dihedral angles”, *J Chem Theory Comput.*, 8, 3257–3273 (2012).

48 W. L. Jorgensen, J. Chandrasekhar, J. D. Madura, R. W. Impey and M. L. Klein, “Comparison of simple potential functions for simulating liquid water”, *J Chem Phys.*, 79, 926–935 (1983).

49 J. B. Klauda, R. M. Venable, J. A. Freites, J. W. O’Connor, D. J. Tobias, C. Mondragon-Ramirez, I. Vorobyov, A. D. MacKerell and R. W. Pastor, “Update of the CHARMM all-atom additive force field for lipids: validation on six lipid types”, *J Phys Chem B.*, 114, 7830–7843 (2010).

50 K. Vanommeslaeghe and A. D. MacKerell, “Automation of the CHARMM General Force Field (CGenFF) I: Bond Perception and Atom Typing”, *J Chem Inf Model*, 52, 3144–3154 (2012).

51 W. Hwang, S. L. Austin, A. Blondel, E. D. Boittier, S. Boresch, M. Buck, J. Buckner, A. Caflisch, H. T. Chang, X. Cheng, Y. K. Choi, J. W. Chu, M. F. Crowley, Q. Cui, A. Damjanovic, Y. Deng, M. Devereux, X. Ding, M. F. Feig, J. Gao, D. R. Glowacki, J. E. Gonzales, M. B. Hamaneh, E. D. Harder, R. L. Hayes, J. Huang, Y. Huang, P. S. Hudson, W. Im, S. M. Islam, W. Jiang, M. R. Jones, S. Käser, F. L. Kearns, N. R. Kern, J. B. Klauda, T. Lazaridis, J. Lee, J. A. Lemkul, X. Liu, Y. Luo, A. D. MacKerell, D. T. Major, M. Meuwly, K. Nam, L. Nilsson, V. Ovchinnikov, E. Paci, S. Park, R. W. Pastor, A. R. Pittman, C. B. Post, S. Prasad, J. Pu, Y. Qi, T. Rathinavelan, D. R. Roe, B. Roux, C. N. Rowley, J. Shen, A. C. Simmonett, A. J. Sodt, K. Töpfer, M. Upadhyay, A. van der Vaart, L. I. Vazquez-Salazar, R. M. Venable, L. C. Warrensford, H. L. Woodcock, Y. Wu, C. L. Brooks, B. R. Brooks and M. Karplus, “CHARMMat45:Enhancements in Accessibility, Functionality, and Speed”, *J Phys Chem B.*, 128, 9976-10042 (2024).

52 B. Roux, “Computational Modeling And Simulations Of Biomolecular Systems”, *World Scientific, Singapore*, (2022).

53 T. A. Darden, A. Toukmaji and L. G. Pedersen, “Long-range electrostatic effects in biomolecular simulations”, *Journal de Chimie Physique*, 94, 1346–1364 (1997).

54 W. Jiang, J. C. Phillips, L. Huang, M. Fajer, Y. Meng, J. C. Gumbart, Y. Luo, K. Schulten and B. Roux, “Generalized scalable multiple copy algorithms for molecular dynamics simulations in NAMD”, *Comput Phys Commun*, 185, 908–916 (2014).

55 J. C. Phillips, D. J. Hardy, J. D. C. Maia, J. E. Stone, J. V. Ribeiro, R. C. Bernardi, R. Buch, G. Fiorin, J. Hénin, W. Jiang, R. McGreevy, M. C. R. Melo, B. K. Radak, R. D. Skeel, A. Singharoy, Y. Wang, B. Roux, A. Aksimentiev, Z. Luthey-Schulten, L. V. Kalé, K. Schulten, C. Chipot and E. Tajkhorshid, "Scalable molecular dynamics on CPU and GPU architectures with NAMD", *J Chem Phys.*, 153(4), 044130 (2020).
